# Reconstruction and logical modeling of glucose repression signaling pathways in *Saccharomyces cerevisiae*

**DOI:** 10.1186/1752-0509-3-7

**Published:** 2009-01-14

**Authors:** Tobias S Christensen, Ana Paula Oliveira, Jens Nielsen

**Affiliations:** 1 Center for Microbial Biotechnology, Department of Systems Biology, Technical University of Denmark, Building 223, DK-2800 Kgs. Lyngby, Denmark; 2 Current address: Department of Chemical Engineering, Massachusetts Institute of Technology, Building 66, 25 Ames Street, Cambridge, MA 02139, USA; 3 Current address: Institute for Molecular Systems Biology, ETH Zurich, CH-8093, Zurich, Switzerland; 4 Current address: Department of Chemical and Biological Engineering, Chalmers University of Technology, SE-412 96, Gothenburg, Sweden

## Abstract

**Background:**

In the yeast *Saccharomyces cerevisiae*, the presence of high levels of glucose leads to an array of down-regulatory effects known as glucose repression. This process is complex due to the presence of feedback loops and crosstalk between different pathways, complicating the use of intuitive approaches to analyze the system.

**Results:**

We established a logical model of yeast glucose repression, formalized as a hypergraph. The model was constructed based on verified regulatory interactions and it includes 50 gene transcripts, 22 proteins, 5 metabolites and 118 hyperedges. We computed the logical steady states of all nodes in the network in order to simulate wildtype and deletion mutant responses to different sugar availabilities. Evaluation of the model predictive power was achieved by comparing changes in the logical state of gene nodes with transcriptome data. Overall, we observed 71% true predictions, and analyzed sources of errors and discrepancies for the remaining.

**Conclusion:**

Though the binary nature of logical (Boolean) models entails inherent limitations, our model constitutes a primary tool for storing regulatory knowledge, searching for incoherencies in hypotheses and evaluating the effect of deleting regulatory elements involved in glucose repression.

## Background

Signaling and regulatory cascades establish the bridge between environmental stimuli and cellular responses, and represent a key aspect of cellular adaptation to different environmental conditions. Cells can sense several stimuli, both internally and externally, and the received information will subsequently be propagated through a cascade of physico-chemical signals. The ultimate recipients of these signals will determine how the cell responds, by acting at different regulatory levels (transcriptionally, translationally, post-translationally, allosterically, etc). Contrary to metabolic networks, most signaling and regulatory pathways are relatively poorly studied and signaling properties of a protein cannot be easily derived from its gene sequence [1,2]. Moreover, signal transduction networks operate over a wide range of time-scales, and due to the presence of feedback loops and cross talk it is difficult to discern how concurrent signals are processed. Thus, methods to analyze and model signal transduction and regulatory circuits are of prime importance in biology, medicine and cell engineering, since they can bring insights into the mechanistic events underlying complex cellular behavior. The availability of models for signaling and regulatory cascades represents an opportunity to expand the search space when looking for intervention targets that may lead to desired phenotypes – e.g. when looking for better drug targets in medicine, designing novel regulatory circuits in synthetic biology or finding regulatory targets that can release metabolic control in metabolic engineering.

**Figure 1:**
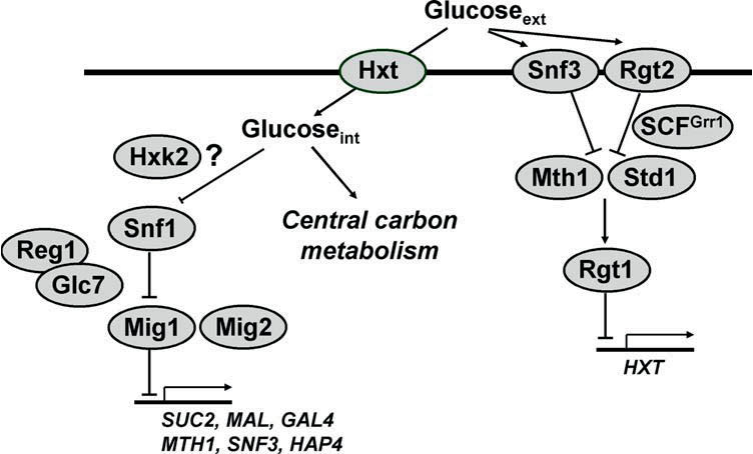
**Simplified representation of the main glucose repression pathways**. Glucose is transported into the cell by hexose transporters with different affinities (*HXT1-HXT16*). Inside the cell, glucose is phosphorylated to glucose 6-phosphate by Hxk2, therefore entering into carbon metabolism. An unknown signal triggered by high glucose levels leads to inactivation of the Snf1 complex. This inactivation is regulated by the protein phosphatase Glc7-Reg1. Inactive Snf1 cannot phosphorylate Mig1, which thus remains in the nucleus under high glucose levels, exerting repression of transcription of several genes. At low glucose concentrations, when Snf1 becomes active, Mig1 is phosphorylated and translocates to the cytosol, releasing repression. Glucose is sensed by two sensors located in the cell membrane, Rgt2 and Snf3. At high glucose levels, the signal from these sensors leads to SCF^Grr1 ^mediated ubiquitination and consequent degradation of Mth1 and Std1, which are required for Rgt1 activation.

Most eukaryotic cells, including many yeasts and humans, can sense the availability of carbon sources in their surroundings and, in the presence of their favorite sugar (often glucose), trigger a cascade of signals that will repress the utilization of less-favorite sugars as well as the function of different catabolic routes [3-5]. This phenomenon is commonly termed carbon-catabolite or glucose repression. Because of its role in nutrient sensing and its industrial impact on the simultaneous utilization of different carbon sources, glucose repression has been a model system for studying signaling and regulation. In particular, glucose repression in the yeast *Saccharomyces cerevisiae *has been extensively studied and two main signaling pathways have been identified: a repression pathway, mediated through the protein kinase Snf1 and the transcription factor Mig1, and a glucose induction pathway, mediated through the membrane receptors Snf3 and Rgt2 and the transcription factor Rgt1 (for review see, for example, [4,6-8]). Growing evidence suggests the existence of extensive cross talking between these two pathways. Figure 1 summarizes key aspects of the system. Besides the role of glucose repression on the utilization of alternative carbon sources, glucose repression in yeast leads to the transcriptional shutdown of genes related to respiration, mitochondrial activities, and gluconeogenesis [9]. This transcriptional behavior causes the wild-type yeast to exert respiro-fermentative metabolism during growth on excess glucose, redirecting carbon towards by-products of metabolism such as ethanol, acetate and glycerol, at the cost of biomass formation. Despite being an extensively studied system, knowledge on yeast glucose repression is still far from complete and key questions remain, including: what exactly triggers the cascade signal(s)? How to differentiate between causes and consequences? How does the knowledge derived from phenotypic observations relate to mechanistic events? How does the current knowledge on glucose repression fit with available high-throughput data? In order to attempt to bring insights into these questions, we aim here at creating a mechanistic, semi-quantitative model of glucose repression signaling cascades and genetic regulatory circuits in yeast.

Modeling approaches of different levels of abstraction have been proposed to analyze and simulate signal transduction and regulatory networks, ranging from purely topological to kinetic models. While attractive in principle, quantitative kinetic models based on ODEs are hampered by difficulties in determining the necessary parameters and kinetic equations. At the other extreme, strictly descriptive models have also been reported [10-12], in which the precision of the formalism proposed, based on process engineering, establish a clear and unique qualitative representation of the network interactions. Somewhere in between lie more semi-quantitative and qualitative approaches that require the topological description of signaling interactions and make use of well-established mathematical frameworks to analyze network structure and functionality. Such methods include (i) stoichiometric representation and extreme pathway analysis [13], (ii) Boolean (on/off) and Bayesian (probabilistic) representation of interactions [14-16], (iii) logical hypergraph representation and logical steady state analyses [17], and (iv) Petri nets graph representation and simulations [18-20]. All these methods describe signal flow qualitatively within a mathematical formalism and without loss of information on the network topology, allowing insightful computations on network structure such as evaluating the degree of cross talk, determining all possible elementary 'flux' modes and calculating the number of theoretically possible positive and negative feedback loops. Moreover, logical hypergraph analyses and Petri net models also have the potential to be used for semi-quantitative simulation of network behavior, since they allow simple predictions of the state of a system in response to different stimuli.

In this work, we reconstructed the signaling and transcriptional regulatory network of glucose repression in *S. cerevisiae *based on *established *knowledge reported in the literature. We converted this information into a logical hypergraph, and performed structural and functional analyses on the network following the framework proposed by Klamt and co-workers [17]. Next, we performed logical steady state analyses to compute the state of all nodes in the system under all possible environmental conditions (sugars availability), and for all different single gene deletions and some double gene deletions. Furthermore, we developed a framework to evaluate model predictions by comparing changes in the state of the regulatory layer against changes in gene expression data (transcriptome data was available for several knockouts of the system). Based on the results from the model evaluation, we identified main errors and discuss possible sources of discrepancies, as well as the inherent limitations to Boolean modeling. Our results point towards the existence of incoherencies between high-throughput data and literature-based knowledge related with glucose repression. To our knowledge, this represents the first attempt to mechanistically and semi-quantitatively model glucose repression signaling and regulatory pathways in the yeast *S. cerevisiae*.

## Results

### Reconstruction of the signaling/regulatory network and model setup

Glucose repression signaling and regulatory network was reconstructed from low-throughput data reported in peer-reviewed publications. Information was gathered based on biochemical studies and physiological observations, and it was included in a collection database containing: (i) list of proteins with sensor, signaling or transcription factor functions found to be related to glucose repression; (ii) list of genes known to be transcriptionally regulated by glucose repression related transcription factors; (iii) type of regulation exerted on each of the previous species by metabolites and/or regulatory proteins. The reconstructed network accounts for 72 species (corresponding to 50 genes) and 148 interactions, which cover most of the current knowledge on the Mig1/Snf1 and Snf3/Rgt2 pathways, as well as galactose and maltose regulatory systems. Transcription factors included are Rgt1, Mig1, Mig2, Mig3, Sip4, Cat8, MalR and Gal4. Regulatory targets include genes encoding hexose transporters and enzymes involved in maltose catabolism, gluconeogenesis and the Leloir pathway. The complete list of species and interactions considered is given as supplementary material (Additional file 1).

**Figure 2:**
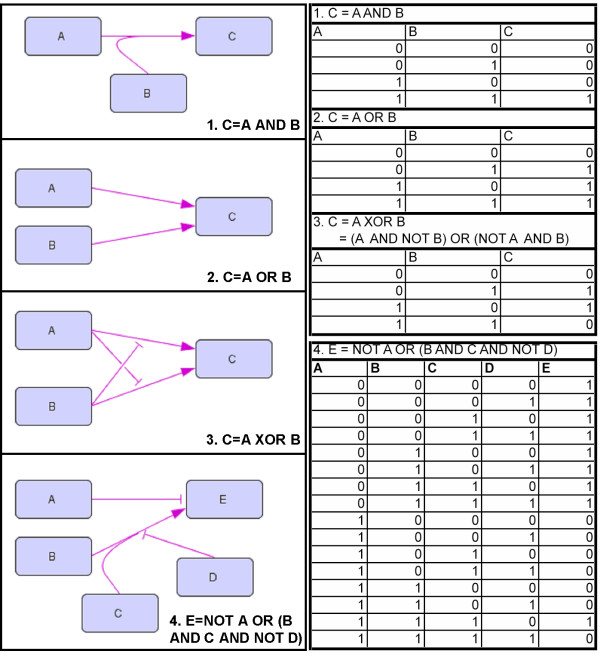
**Example of Boolean expressions and corresponding logical hypergraphs and truth tables**. A logical hypegraph is an interaction network where each edge (or hyperarc) connects a set of start-nodes (tails) to an end-node (head), and the combination of incoming hyperarcs to an end-node represents a Boolean expression. Any Boolean expression can be written in a disjunctive normal form (only using AND, OR and NOT operators). On the disjunctive normal form, expressions are built up by literals (i.e. variables or their negation) connected by AND relations forming clauses, and clauses can then be connected by OR relationships. In the logical hypergraph, each clause is represented by a hyperarc, while separate hyperarcs linked to an end-node represents clauses connected by an OR relationship. When the value of a tail species is negated, this is marked by a repression symbol in the corresponding hyperarc (see also the symbolic explanation in Figure 3). The Boolean function determining the state of a node is thus given by all the incoming hyperedges.

**Figure 3:**
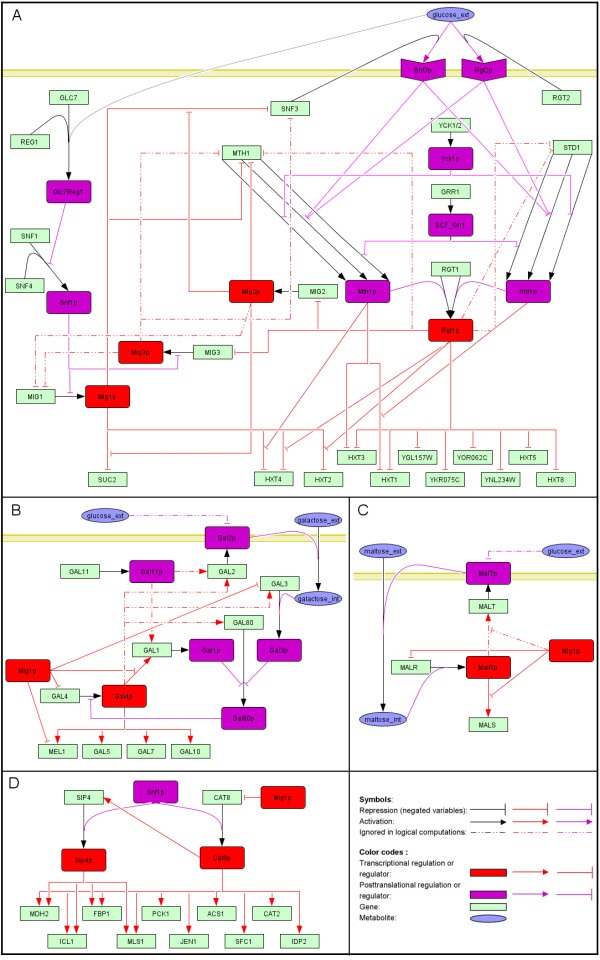
**Hypergraph representation of the Boolean model for yeast glucose repression**. A) Overall representation of the main glucose repression pathways, including the Snf1/Mig1 repression pathway and the Snf3/Rgt2 induction pathway. Note how the high degree of cross talk makes it impossible to distinguish between the two pathways. B) The GAL regulatory system. C) The MAL regulatory system. *MALT *is set to be active by default, since MalT is assumed to be present at a basal level. D) Subset of the network controlled by Sip4 and Cat8. Signaling proteins are represented with purple rectangles, while transcription factors are represented with red rectangles. Accordingly, a post-transcriptional regulatory association is represented with a purple line, while a transcriptional regulatory interaction is represented with a red line. Gene transcripts are represented with green rectangles, and sugar metabolites are depicted by blue ellipses. The description of the representation of logical relations between species is given in the legend of Figure 2. A note about the nomenclature in the hypergraph representation: genes are denoted in uppercase (e.g., MIG1), while protein names include the suffix 'p' (e.g., Mig1p). In the main text, proteins are named similarly, but without the suffix (e.g., Mig1), and genes are in italic uppercase (e.g., *MIG1*)

Thereafter, the reconstructed signaling/regulatory network was converted into a logical hypergraph (Figure 2), representing all interactions in a logical manner (Figure 3 and Additional file 2), according to the framework proposed by Klamt *et al*. [17] to model signaling networks. The conversion of signaling and regulatory interactions into Boolean functions was based on described functions reported in the literature (the rationale for the choice of less obvious Boolean functions for certain interactions is explained in the Additional file 2). The resulting hypergraph consists of 77 nodes (50 genes, 22 proteins, 3 extracellular metabolites and 2 intracellular metabolites) and 118 hyperedges, and represents a logical model for glucose repression signaling and regulatory pathways. For ease of visualization, we have depicted the hypegraph into four separate sub-networks, each representing in more detail a different system: Mig1/Snf1 and Snf3/Rgt2 pathways (Figure 3A), galactose regulation (Figure 3B), maltose regulation (Figure 3C) and the Sip4/Cat8 regulatory system (Figure 3D). The model takes as input the availability of carbon sources (glucose, galactose, and maltose) and outputs the logical steady state of the network.

In our model, nodes can assume one of two logical states, 1 or 0, corresponding to on or off, or in more subtle instances, higher or lower activity. For protein nodes, this can most simply be interpreted as a protein being active (1) or inactive (0), whereas in the case of gene nodes, it can be seen as a gene being expressed (1) or not (0). We used the model to analyze structural characteristics of the network and to compute logical steady states of all nodes in the network. In particular, we simulated how gene transcripts change their logical state in response to perturbations (e.g., availability of different sugars and different gene knockouts), and evaluated the predictions by using available transcriptional datasets for different carbon source conditions and yeast deletion mutants. During logical states simulations, although most nodes were left unconstrained, a few nodes were assigned a default value of 1. E.g., *GRR1 *was set to a fix state of 1 since its regulation is not considered in the model (otherwise, no unique logical steady state would exist). Also, in the case of genes constitutively expressed at a basal level such as *MALT*, encoding a maltose transporter, the node state was set to a fix value of 1.

### Structural and functional analyses of the network

Logical steady state analyses were performed for all combinations of sugar availability for the wild type, all single gene knockout mutants (24 cases), and three double gene deletion mutants of interest, in a total of 224 simulations (see Additional file 3). We notice that most of the gene nodes change their logical state in over 10% of the simulations, but only few (*MTH1*, *MALR*, *GAL3*, *GAL4 *and *CAT8*) change in more than 15% of the simulations. The predictions for the wild type along with a subset of the deletion mutants for which transcriptome data was available were analyzed and used to evaluate the model, as will be discussed below. The capability to make semi-quantitative predictions of gene expression levels and protein activity for any combination of gene deletions and nutrient conditions is a key feature of the model.

**Figure 4:**
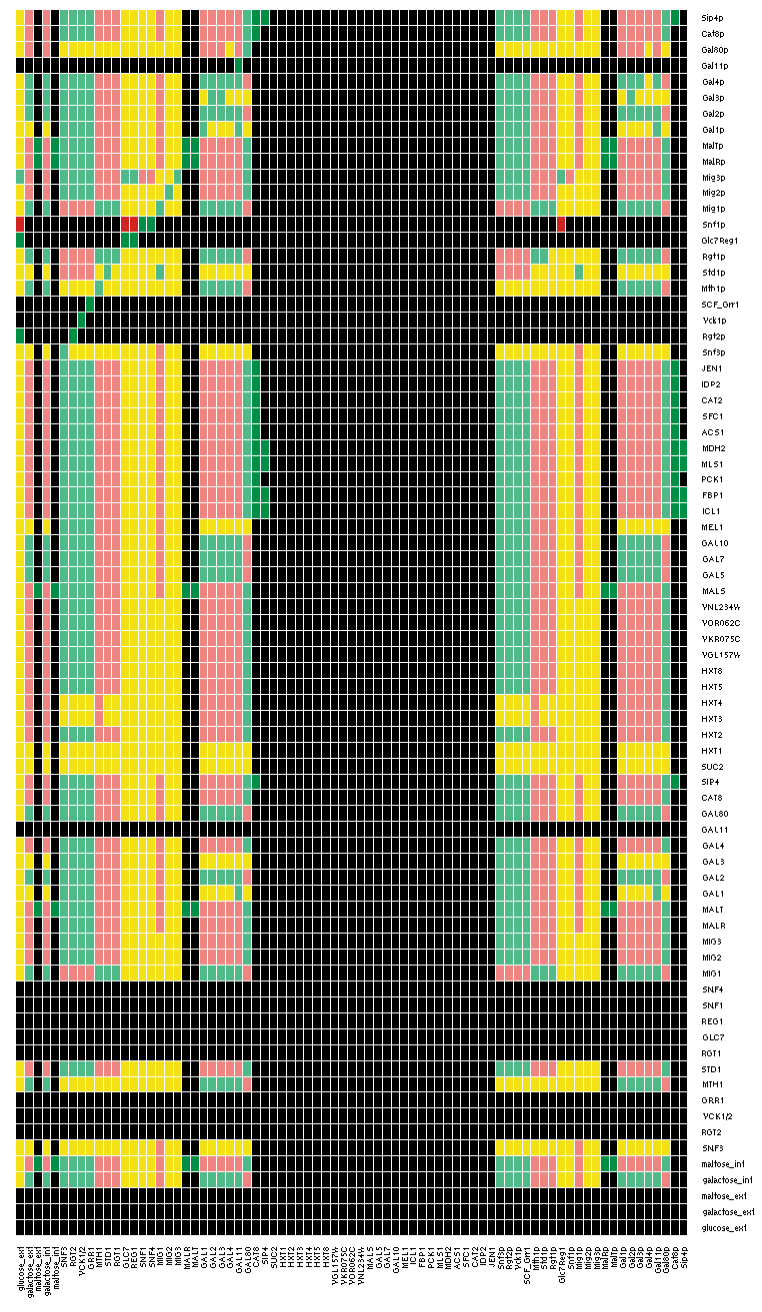
**The dependency matrix for the yeast glucose repression network**. Each element in the matrix shows the relationship between an effecting species and an affected species, specified at the bottom of the column and at the end of the row, respectively. A yellow field in the intersection of the i^th ^column and j^th ^row signifies that the i^th ^species is an ambivalent factor with respect to the j^th ^species, i.e. that both activating and repressing/inhibiting paths from the i^th ^to the j^th ^species exist. For example, the yellow color of the first element (first column and first row) indicates that both inhibiting and activating paths exists from exterior glucose to the Sip4 protein, i.e., exterior glucose is an ambivalent factor with respect to Sip4. Similarly, a dark green field and a light green field indicate a total and a non-total activator, respectively, i.e., only activating paths exist and negative feedback loops are either absent (total) or present (non-total). Dark red and light red fields represent total and non-total repressors/inhibitors, respectively. A black field indicates that no path exists from A to B. The large number of black columns in the middle corresponds to output species (sinks), which per definition are non-affecting towards all species. Due to the directional nature of the interaction network, the matrix is not symmetric (e.g. Sip4 is non-affecting toward exterior glucose). See nomenclature note for gene and protein species in the legend of Figure 3.

The reconstructed network contains 35 negative and 14 positive feedback loops, which is indicative of the high degree of crosstalk between pathways and complex cause-effect relationships. This is further supported by the dependency matrix of the network (Figure 4), which is based solely on the underlying network interaction graph without information on Boolean functions. In the matrix, yellow elements represent an ambivalent relationship between an ordered pair of species (*i,j*), where *i *and *j *represents the column and the row number, respectively, in the sense that both activating and inhibiting paths exist from *i *to *j*. Dark or light green (/red) elements, D_ij_, indicate that species *i *is a total or non-total activator (/inhibitor) of species *j*, respectively, i.e. only activating (/inhibiting) paths from *i *to *j *exist and feedback loops are either absent (total) or present (non-total) – see Figure 4 legend for details. Examining the dependency matrix, the large number of ambivalent relationships (represented by yellow elements) as well as the prevalence of negative feedback loops in signaling paths (light green or red fields) is noteworthy. It underscores the difficulties in making predictions based on intuitive approaches and emphasizes the need for a logical modeling framework. The matrix also reveals the high degree of crosstalk between the Mig1/Snf1 and Snf3/Rgt2 pathways, since it is quickly noted that the signaling proteins in one pathway generally affect proteins in the other pathway.

### Model evaluation

In order to evaluate the capability of the logical model to predict differential gene expression, we performed logical steady state analysis of the glucose repression regulatory response for five different gene knockouts, and compared the results with available whole-genome gene expression from DNA-microarrays. We used data from the yeast mutants *Δrgt1*, *Δmig1*, *Δmig1Δmig2*, *Δsnf1Δsnf4*, *Δgrr1 *and their isogenic reference strains. This type of analysis not only gives an indication of the model's predictive strength, but also hints at possible errors in the model (and eventually in the underlying hypotheses from the literature) in the cases where discrepancies between model and observation occur.

The best we can hope to achieve with a Boolean model is a correct prediction of the sign of the change in gene expression, i.e., the model prediction Y_i_^mod ^should equal the experimental observation Y_i_^exp ^when evaluating change in expression of gene *i *following a knockout or change in conditions. In order to assess what experimental results should be regarded as *a change*, it is necessary to make an interpretation of the gene expression data that allows a comparison with the binary outputs of the Boolean model. Intuitively, the experimental change should be relatively large and statistically significant in order to be reflected by the discrete Boolean model. Therefore, we established a fold-change threshold ([FC_min_| = 1.5; see Methods for fold-change definition) and a Student's *t*-test *p*-value cut-off (α = 0.05) for all pairwise gene expression comparisons between a deletion mutant and its isogenic reference strain. All genes with *p*-value < α and FC ≥ FC_min _(or FC ≤ -FC_min_) were assigned with a value of Y_i_^exp ^= 1 (or Y_i_^exp ^= -1), and 0 otherwise. Such conversion of gene expression into discrete Boolean values based on a somewhat *subjective *threshold may yield a number of type-2 (false negatives) and type-1 (false positives) errors.

The hereby identified experimental variation, Y_i_^exp^, was then compared with the model prediction, Y_i_^mod^, for each gene *i*. Y_i_^mod ^was determined by the difference in Boolean output for gene *i *between the mutant and the reference state (wildtype), at a defined external condition. Thus, Y_i_^mod ^can assume the values -1, 0 or 1, corresponding to a decrease, no change or increase in gene expression on transcript *i *in the mutant, respectively. Model prediction capabilities were evaluated based on the difference |Y_i_^mod ^- Y_i_^exp^|, with a value of 0 meaning a correct prediction, a value of 1 implying a small error, and a value of 2 indicating a large error (only happening when model prediction and experimental results point towards opposite directions).

A summary of the results from the comparison between model prediction and experimental up- and down-regulation for all five different knockouts evaluated is shown in Table 1. In the following, we discuss more thoroughly the results for *Δrgt1*, and use this to analyze common reasons for discrepancies in all the knockouts. The remaining comparisons are briefly commented afterwards.

**Table 1:** Summary of results from the model evaluation.

**Knockout Genotype**	**Number of Genes Evaluated**	**Number of true predictions**	**Percentage of true predictions for genes changed on array**	**Percentage of true predictions for genes changed in the model**	**Percentage of true predictions**
***Δrgt1***	34 (25)	28 (21)	83% (83%)	71% (63%)	82% (84%)

***Δmig1***	38 (27)	24 (20)	29% (44%)	40% (57%)	63% (74%)

***Δmig1Δmig2***	38 (28)	24 (19)	47% (53%)	69% (80%)	63% (68%)

***Δsnf1Δsnf4***	34 (25)	17 (14)	45% (60%)	56% (64%)	50% (56%)

***Δgrr1***	38 (28)	15 (10)	25% (24%)	55% (100%)	39% (43%)

In parentheses are the numbers when dubious genes are not included in the computations (see Discussion for details).

#### Evaluation of Δrgt1 mutant

Transcriptome data for the yeast *Δrgt1 *mutant and its isogenic reference strain is available from [21] during shake flasks cultivations using galactose as the single carbon source. Sampling was made in the mid-exponential phase, where pseudo-steady-state can be assumed (i.e., growth rate and physiological yields appear constant during the exponential phase, despite changes in the concentration of extracellular metabolites). We converted the gene expression data from this study into Y_i_^exp ^according to the procedure described above, and compared it with the simulation results Y_i_^mod ^(Table 1). Y_i_^mod ^are derived from logical steady state analyses of the logical model assuming galactose present and all other carbon sources absent. The logical steady states were first determined for the original model without further constrains (X_i,WT_). Afterwards, in order to simulate the *RGT1 *gene deletion, the node RGT1 of the hypergraph was set to zero, and a new logical steady state analysis was performed (X_i,RGT1_). Y_i_^mod ^is given by the difference between X_i,RGT1 _and X_i,WT_.

In general, the model predictions are very good for the *Δrgt1 mutant*, with 82% true predictions, reflecting the fact that Rgt1 along with its regulators has been extensively studied [21-30]. Only for 6 out of the 34 genes evaluated, the experimental fold changes do not correspond to model prediction. The six genes are *SNF3*, *MTH1*, *MIG2*, *MAL*33 (which encodes a *MAL *regulator), *SUC2 *and *HXT8*. In the following, the causes of these discrepancies are investigated.

***Analysis of discrepancies in SNF3. ****SNF3 *encodes a high-affinity glucose sensor located in the plasma membrane. Gene expression data shows no differential expression of *SNF3 *in the *Δrgt1 *mutant (*p*-value = 0.88, FC = 1.03). However, in the model this gene is found to be down-regulated in the mutant relative to the wild type. The most likely explanation for this discrepancy lies in the logical equation for *SNF3 *applied in the model:

*SNF3 *= NOT (Mig1 OR Mig2) = (NOT Mig1) AND (NOT Mig2)

In the model, *RGT1 *deletion leads to an active Mig2 and consequently repression of *SNF3*. The most plausible explanation for this is that the model overestimates the importance of Mig2 and that, in reality, the presence of active Mig2 by itself is not enough to prevent *SNF3 *transcription. Nevertheless, this explanation cannot be accepted out of hand: the dependency matrix reveals that *RGT1 *is an ambivalent factor with regard to *SNF3*, i.e., both repressing and activating paths from *RGT1 *to *SNF3 *exist. It is thus possible that Mig2 is indeed a significant repressor of *SNF3*, but that other important, repressing pathways from *RGT1 *to *SNF3 *are also deactivated by the *RGT1 *deletion.

A closer inspection of the signaling paths from *RGT1 *to *SNF3 *in the hypergraph reveals that, from the 8 existing possible paths, 4 are repressing paths of the form *RGT1 *-> Rgt1 -| Mig2/Mig3 -| Mig1 -| *SNF3*. This means that Rgt1 represses Mig2 and Mig3, both of which repress Mig1, which then represses *SNF3 *(this constitutes 4 paths because two hyperedges connect *RGT1 *to Rgt1, either with Mth1 or Std1 as the second tail). It seems unlikely that these 4 signal flux modes play significant physiological roles since (i) no significant role of Mig3 has ever been found [31-33], and (ii) Mig2 can at most serve to attenuate Mig1 expression since the two proteins are active at basically the same conditions.

***Analysis of discrepancies in MTH1.*** Mth1 is a signaling protein intermediate between the membrane sensors Snf3/Rgt2 and the transcription factor Rgt1 [23,24]. Gene expression of *MTH1 *is found to be up-regulated in the *Δrgt1 *mutant, whereas the model predicts the expression level of *MTH1 *to be unchanged. Despite the fact that Rgt1 repression of *MTH1 *is reported in the literature (in fact in the same paper where *Δrgt1 *transcriptome data is presented) [21], the repression of *MTH1 *by Rgt1 is ignored for logical steady state calculations for two reasons. Firstly, Kaniak *et al*. state that this transcriptional repression is weak (the transcriptome analysis was actually complemented with promotor-lacZ fusions, and it was found that *MTH1*, both in the wild-type and in the *Δrgt1 *mutant, is subject to considerable glucose repression, not glucose induction as would be expected for a gene primarily regulated by Rgt1 [21]. Secondly, in terms of the Boolean model, including Rgt1 in a "NOT Rgt1 AND-relationship" would mean that Mth1 would always be inactive when Rgt1 is active, which would be somewhat incongruous considering that *MTH1 *seems to encode a co-repressor of Rgt1. This example illustrates the difficulties in incorporating negative feedback loops in a binary Boolean model.

It should also be noted that *MTH1 *is, according to the model at least, one of the most heavily regulated genes in the network, being transcriptionally regulated by Rgt1, Mig1, Mig2, Mig3 and Gal4. This makes a literature based determination of the Boolean function governing its expression particularly difficult.

***Analysis of discrepancies in MIG2 and HXT8.**** MIG2 *encodes for a homologue of the transcription factor Mig1, while *HXT8 *encodes for a plasma membrane hexose transporter. *MIG2 *was not found to change experimentally, at least not in terms of our defined "Boolean fold change" threshold, but was found to be up-regulated in the model. This discrepancy is, most likely, due to a type-2 error in the inference of *gene expression change *from the transcriptome data. Even though the average fold change observed experimentally was 3.4, the *p*-value of this change was only 0.29. As this is above the cut-off value of 0.05, this change is deemed insignificant and the gene is attributed a "Boolean fold change" of 0. Nevertheless, the model prediction is in good agreement with the results of the continued investigation by Kaniak *et al*., which included promoter-lacZ fusions and ChIP experiments, and showed that Rgt1 is a strong (and possibly the only) transcriptional repressor of *MIG2*. The discrepancy for *HXT8 *seems to have similar reasons.

***Analysis of discrepancies in MAL33 (MALR).*** Expression of *MAL*33, encoding a *MAL *regulator, was found to be experimentally repressed in the mutant, while it remained unchanged in the model. The three genes required for maltose metabolism are mapped in various *MAL *loci, of which 5 are currently known [34,35]. In the model, no distinction is made between the different complex loci. Since this is a recurrent discrepancy in all evaluations performed, we present a general discussion on the *MAL *regulatory system later in the Discussion section.

***Analysis of discrepancies in SUC2.*** Expression of *SUC2*, encoding for invertase (sucrose hydrolyzing enzyme), was not found to be significantly different between the *Δrgt1 *mutant and wild-type, whereas the model predicts a decrease in gene expression in the mutant. This discrepancy illustrates both the difficulties in choosing the correct logical equation for a specific species based on literature and in converting a continuous reality to a binary model.

In the model it is assumed that both Mig1 and Mig2 should be absent in order for *SUC2 *to be expressed at high levels (SUC2 = NOT (Mig1 OR Mig2)), cf. Lutfiyya *et al*. who found that the single deletions had relatively low impact on *SUC2 *expression level, whereas *Δmig1Δmig2 *double deletion had great effect [36]. Contrary to this, Klein *et al*. found a large increase in *SUC2 *expression in a *Δmig1 *strain and further increase by additional disruption of *MIG2 *[37]. The Klein *et al*. observations could have been implemented in the model via a Boolean function where the absence of either one of the two repressors induces expression of *SUC2 *(i.e., SUC2 = NOT (Mig1 AND Mig2)) or, alternatively, by simply ignoring Mig2 in the equations. While implementing either of the alternatives would have led to correct model prediction for the knockout (as judged by comparison with the expression data), the actual output for *SUC2 *in the wild-type would have been the same regardless of the chosen equation (since absence of Mig 1 or of both Mig1 and Mig2 would always result in active *SUC2*). Nevertheless, the ambiguity in the literature combined with the difficulty of imposing a discrete model on a continuous reality made the choice of logical equation extremely hard in this case.

The model apparently mistakenly predicts a decrease in *SUC2 *expression, because *RGT1 *deletion causes *MIG2 *to be expressed (i.e. Mig2 becomes active) which then leads to repression of *SUC2 *with the chosen Boolean equations. Based on this evaluation, and assuming that a type-2 error has not occurred, it therefore seems reasonable to say that the model overestimates the importance of Mig2 in the regulation of *SUC2*. Alternatively, it is possible that *MIG2 *transcription is induced, but that the Mig2 protein is also post-translationally regulated, something that is not described in the model or, to the best of our knowledge, in the literature. This could mean that Mig2 protein activity is not increased by the deletion of *RGT1*, despite the eventual increase in transcript levels.

#### Evaluation of Δmig1 and Δmig1Δmig2 mutants

Transcriptome data for *Δmig1 *and *Δmig1Δmig2 *mutants, and their isogenic reference strain was available from [38] during aerobic batch cultivations using 40 g/L glucose as single carbon source. Samples were taken in the mid-exponential phase at a residual glucose concentration of 20 g/L. Therefore, experimental observations were evaluated against model predictions with only glucose present. For both knockouts, the percentage of true predictions is of 63% (Table 1). When examining the discrepancies between experimental observations and model predictions, it is particularly noticeable that model predictions for *MAL *and *GAL *regulatory genes are almost always wrong. In particular, in the *Δmig1 *case both *MAL13 *and *GAL4 *evaluation produces a large error (i.e., |Y_i_^mod ^- Y_i_^exp^| = 2), meaning that the experimental direction of regulation is opposite to that predicted by the model. Whereas in the case of *MAL *genes this is one of several discrepancies observed in this work (see Discussion), in the case of *GAL4 *we find the experimental gene expression data from [38] to directly contradict the results of a Northern blot analysis [39]. While the former study showed a decrease in *GAL4 *levels upon deletion of *MIG1*, the second showed more than 6-fold increase in the levels of *GAL4 *transcripts in a *Δmig1 *mutant, in a medium with the same glucose concentration (as cited in [40]).

#### Evaluation of Δgrr1 mutant

Transcriptome data for *Δgrr1 *and its isogenic reference strain were also available from [38], at conditions corresponding to high extracellular glucose concentration. Evaluation of model predictions for *Δgrr1 *yielded the poorest results, with only 39% of true predictions (Table 1). This is probably due to the fact that, despite being an important player in a large number of cellular activities such as cell morphology, heavy metal tolerance, osmotic stress and nitrogen starvation [25,30], Grr1 is in the model only represented as a simple regulator acting upstream of Rgt1. The highly pleiotropic nature of Grr1 is revealed in the DNA-microarray data, where 24 out of 38 evaluated genes are found to be differentially expressed (in the Boolean sense). The low percentage of correct predictions illustrates the danger of including species without properly accounting for all key functions. We notice that the model even fails to efficiently predict the alteration of gene expression for targets of Rgt1 (e.g. *HXT2*, *HXT4*, *HXT5 *and *HXT8*), which, according to the model, should act downstream of Grr1. However, given the scale of the perturbations caused by *GRR1 *deletion, it is difficult to say whether these discrepancies are caused by faults in the current hypotheses underlying the model or by secondary effects not taken into consideration, such as altered metabolism.

#### Evaluation of Δsnf1Δsnf4 mutant

Transcriptome data for *Δsnf1Δsnf4 *and its isogenic reference strain were available from [41], during aerobic continuous cultivations at dilution rate 0.1 h^-1 ^and glucose (10 g/L) as single carbon source. Under these conditions, the residual glucose concentration inside the fermentor is very low, and typically no glucose repression is observed. This behavior is, to some extent, similar to what happens in the absence of glucose. Thus, experimental observations were compared with model predictions for the case where all sugars were absent. The percentage of true predictions was only 50% (Table 1). A surprising discrepancy was observed for the expression levels of *CAT8 *and *SIP4*, which were predicted to decrease, but are found to be experimentally unchanged (in the Boolean sense). Nevertheless, the prediction of a down-regulatory effect on Cat8 and Sip4 gene targets (*ICL1*, *FBP1*, *PCK1*, *MLS1*, *MDH2*, *ACS1*, *SFC1*, *CAT2*, *IDP2 *and *JEN1*) is confirmed experimentally. While the observed changes for *CAT8 *and *SIP4 *are not statistically significant, they are nevertheless in the predicted direction, and the occurrence of type-2 errors is therefore likely. An additional and likely explanation is that the effect of the Snf1-Snf4 complex on repression by Sip4 and Cat8 is, to a larger extent, mediated via direct posttranslational regulation rather than via indirect, Mig1 mediated transcriptional regulation.

### Global evaluation of predictive power

Boolean models lie at the boundary between qualitative and quantitative models. For the present model of glucose repression, testing current hypotheses is at least as important as making predictions. These goals, however, are connected in the sense that one way in which inconsistencies in the model (and consequently, in the hypotheses proposed in the literature) are revealed is through failure to make predictions. Therefore, evaluating the predictive power of the model is an important task. Above, we looked into the capacity of the model to predict differential gene expression for individual knockouts, and observed that the frequencies of correct predictions varied between the different knockouts (and in no case were all predictions true). What remains to be demonstrated is that the correct predictions were not obtained by chance.

For the simplest random model, the probabilities that the expression of a gene is predicted to be unchanged, up-regulated or down-regulated, respectively, would all equal 1/3. Overall, the average probability of having the correct prediction in each situation would also be 1/3. Table 1 shows that, for all knockouts, the frequency of correct predictions exceeds 33%. We tested the significance of this occurrence by applying a binomial distribution test and p_0 _= 0.33 (cf. Methods) to all predictions (Table 2). Overall and for most knockouts, the very low *p*-values unambiguously show that the results were not obtained due to chance. However, for *Δgrr1 *it cannot be shown at 95% confidence level that the prediction rate is significantly better than with a random model.

**Table 2:** Statistical evaluation of the significance of the model prediction

H_0_: X/N = 1/3 H_1_: X/N > 1/3	***Δrgt1***	***Δmig1***	***Δmig1Δmig2***	***Δsnf1Δsnf4***	***Δgrr1***	**Overall**
***p*-value**	6.7 × 10^-10^	4.8 × 10^-5^	4.8 × 10^-5^	1.2 × 10^-2^	2.1 × 10^-1^	2.6 × 10^-14^

*P*-values for the statistical evaluation of the null hypothesis, H_0_, tested for whether the observed level of success was due to chance, i.e. that the average proportion of correct predictions (X/N) is 1/3, against the alternative hypothesis that the model yields a higher proportion of true predictions. The test is based on the binomial distribution (cf. Methods)

Next, we applied the same distribution to test the significance of the fraction of large errors observed (i.e., |Y_i_^mod ^- Y_i_^exp^| = 2) in the subset of cases where differential expression was observed experimentally as well as in the model. Since in this case there would be 50% chance of a correct prediction if nothing was known *a priori*, we use p_0 _= 0.5. Out of 41 cases, only 3 cases of opposite signs predictions are observed. This is significant with a *p*-value less than 10^-90^. The three encountered large errors were in two predictions for *MALR *(discussed below) and one prediction for *GAL4 *in the *Δmig1 *mutant (most likely arising from the microarray experiment itself, as discussed above).

## Discussion

The goal of our Boolean network modeling and analyses was dual. First, we wanted to use the model to test how the underlying biological hypotheses (for signal transduction and transcriptional regulation) found in literature fit the observations from genome-wide gene expression studies of different signaling knockout mutants. We proposed a framework to compare simulation results with experimental observations and looked for discrepancies between the two. These discrepancies hinted at the identification of different types of errors, as discussed below. Second, we wanted to evaluate the model's predictive strength and investigate to what extent we could use it to simulate transcriptional responses upon deletion of various components of the glucose repression cascade. Such a model can eventually be combined with genome-scale stoichiometric metabolic models to further constrain the solution space during optimization problems (e.g., flux balance analysis). Gene expression data in the form of discrete Boolean on/off information has been previously used to constrain fluxes (or more precisely, the genes encoding the enzymes catalyzing the corresponding reactions) in stoichiometric metabolic models, adding a layer of transcriptional regulation into this type of models [42,43]. Our approach goes even further by also including signaling information, opening new doors to search for targets that can release metabolic control at different regulatory levels.

Glucose repression is a complex and intertwined regulatory system, with extensive cross-talk among pathways, feedback loops and different levels of regulation responding at different time scales. This makes it difficult to decide the logical rules describing some of the species and their influence, in particular species that are heavily regulated (e.g., Mth1) or those with extensive pleiotropic effects (e.g., Grr1). Noticeably, we explicitly decided not to include hexokinase-2 (Hxk2) in the model, despite Hxk2 being hinted to be a key regulator in the Snf1/Mig1 pathway (in addition of its role as a glycolytic enzyme). This decision was based on observations that changes in the activity of Hxk2 lead to an array of effects, namely altered metabolism, which may indirectly trigger other regulatory responses [38,44]. Thus, it becomes difficult to describe the role of Hxk2 in a model of moderate size like ours. More knowledge on the exact signaling and regulatory roles of Hxk2 in glucose repression will be necessary before it can be included in our model.

We performed logical steady state analyses for the wildtype, all single gene deletions and three double gene deletions under all combinations of sugar availability, and observed that most nodes change their logical steady state in more than 10% of the gene deletion simulations. Thereafter, we evaluated the model predictions against available gene expression data by comparing changes in transcript levels (converted into a Boolean form) with simulations of the logical steady state of the model. Determination of the overall true prediction rate for the analyzed knockouts shows that *Δrgt1 *yielded the best results (82%), while true predictions for *Δgrr1 *were the weakest (only 39%). The highest prediction rate found for *Δrgt1 *is probably related with the fact that many of the regulatory mechanisms included in our model are originated from transcriptional studies on the role of Rgt1. Conversely, the very bad prediction capabilities for *Δgrr1 *are likely related with the pleiotropic role of Grr1 in nutrient sensing, and the fact that our model does not account for all the regulatory effects associated with Grr1. Somewhere in between, true predictions for *Δmig1*, *Δmig1Δmig2 *and *Δsnf1Δsnf4 *lay in the range 50%–63%. These prediction rates can be improved if we do not account for genes with dubious regulation. Namely, the genes *HXT5*, *HXT8*, *YGL157W*, *YKR075C*, *YOR062C*, *YNL234W *and the *MAL *loci were included in the model even tough little is known about their regulation. These genes are presumably regulated by one or two of the transcription factors in the model via a hypothesized mechanism (see Additional file 1), but may very well also be regulated via other mechanisms, possibly involving other regulators. Nevertheless, their inclusion in the model allows us to see to what extent their expression is explained by the proposed mechanism. We observed that, if these dubiously regulated genes were not included for model evaluation, the rate of correct predictions increased markedly in the case of *Δmig1*, *Δmig1Δmig2 *and *Δsnf1Δsnf4 *(see values in parenthesis in Table 1). Moreover, if we exclude the genes with dubious regulation mentioned above as well as the results from the highly pleiotropic knockout of *GRR1*, we observe an improvement in the overall success rate from 60% to 71%. This suggests that the proposed mechanisms are probably incomplete for these genes.

Overall, analyses of the discrepancies between model predictions and transcriptome data hints at four main sources of errors: (i) errors arising from imprecise conversion of knowledge into logical representation, (ii) errors inherent to the Boolean formalism, (iii) errors arising from the discrete evaluation of experimental gene expression data, and (iv) situations where high-throughput data goes against literature-based knowledge.

***Errors arising from imprecise conversion of knowledge into logical representation.*** Many of the discrepancies found in the model are originated from biological ambiguities or from difficulties in translating biological behavior into logical rules. For example, during evaluation of the *Δrgt1 *results we saw that it was not trivial to describe in Boolean terms the regulation of *SNF3 *and *SUC2 *by Mig1 and Mig2. In both cases, the error seems to arise from an over-estimation of the importance of Mig2 repression. Although Mig1 and Mig2 are believed to have somewhat redundant roles as repressors, the Boolean formalism does not make it easy to distinguish different levels of regulation. Most notoriously, the model fails to predict the response of the *MAL *genes (only 1/3 of the predictions were correct, which is the same frequency expected for a random model). In *S. cerevisae *the *MAL *genes, that are required for utilization of maltose as carbon source, co-locate in telomere-associated *MAL *loci. Although there are five different *MAL *loci identified [45] there is large variations in terms of presence and activity of these loci in different strains [35]. Moreover, experimental studies suggest that different *MAL *loci are regulated by distinct regulatory mechanisms, and, in particular, the *MAL6 *locus has been reported to be regulated differently than the *MAL1*, *MAL2 *and *MAL4 *loci [45,46]. In the present work, network reconstruction of the MAL system was based on investigations of the *MAL*6 locus [34,45,47]. However, during model evaluation we made no distinction between different alleles – e.g. *MALR *is used to represent all *MAL *activator encoding genes, i.e. *MAL*13, *MAL*23, *MAL*33, *MAL*43 and *MAL*63 (cf. [48]). This generalization may be a major reason for the very low prediction rates.

***Errors inherent to the Boolean formalism.*** Another source of discrepancies is the limited nature of the binary Boolean model itself. In some cases, very steep response curves for gene expression and protein activities are observed, corresponding well with the binary nature of the Boolean model. However, the Boolean formalism lacks the capacity to describe a continuous reality that cannot be represented in an on/off manner. For example, it is impossible to distinguish between absence, low levels and high levels of glucose, three different conditions that trigger different regulatory responses. Thus, a discretionary approximation that conveniently explains the biological context has to be made (e.g., for the *Δsnf1Δsnf4 *evaluation, low levels of glucose in a carbon-limited chemostat were approximated by a situation of absence of glucose). In other instances, the Boolean formalism may not be sensitive enough to represent different levels of expression, such as in the case of regulation of *GAL1 *by Mig1, Gal4 and Gal80 [49,50]. Furthermore, when considering inducible proteins that are expressed at a basal level, a value of zero may indicate presence at basal level rather than total absence. In such instances, defining whether a gene is being expressed (1) or not (0) is somewhat subjective. A further limitation of the Boolean formalism, particularly when focusing on logical steady state analysis with no distinction between processes of different time scales, is the difficulty of incorporating negative feedback loops. As discussed above there are difficulties in representing the negative feedback loop regulating *MTH1 *expression, and it is also impossible to represent Mig1 repression of the *MIG1 *gene. Such auto-repression cannot be included by an AND-relationship, since *MIG1 *(and Mig1) would then never be active in a logical steady state.

***Errors arising from the discrete evaluation of experimental gene expression data.*** Conversion of gene expression changes into discrete Boolean values is a simplification, which is presumably prone to errors. Nevertheless, we used commonly accepted thresholds of fold-change and significance to decide whether a gene is changing its expression. We have also checked whether choosing different α and FC_min _greatly impacts model evaluation results (results not shown), and observed that it does not. We have identified a number of discrepancies likely to be due to type-2 errors when assigning experimental variation (e.g., *MIG2 *and *HXT8 *in the *Δrgt1 *case, and *CAT8 *and *SIP4 *in the *Δsnf1Δsnf4 *case), which show a regulatory change in the expected direction although statistically is not significant. However, one must look into these errors carefully, especially when the significance of the experimental change is calculated based on a high number of replicates, since this may hint at an error in the underlying hypothesis instead of a type-2 gene expression error.

***Situations where high-throughput data goes against literature-based knowledge.*** Large errors found in the model evaluation (|Y_i_^mod ^- Y_i_^exp^| = 2) indicate situations where model prediction and observed changes of gene expression have opposite signs. This type of error represents a situation where the hypothesis underlying the logical model needs to be reconsidered. We have found this type of error only 3 times (for *GAL4 *and *MAL13 *in *Δmig1*, *MAL33 *in *Δsnf1Δsnf4*). In the case of the *MAL *genes, we have already discussed probable sources of the wrong predictions, namely the incorrect assumption about the regulatory mechanisms controlling the expression of the *MAL *loci. For the *GAL4 *gene, we have observed that gene expression from the *Δmig1 *transcriptome study directly contradicts other studies. Although more careful analysis may be advisable, it is likely this result arises from the microarray experiment itself, either due to a problem with the array hybridization or with the normalization method used at probe-sets level.

## Conclusion

Overall, the Boolean model showed some potential as a predictive model. The overall success rate (60% for the entire model, 71% for the restricted model without genes of dubious regulation and without considering deletion of highly pleiotropic *GRR1*) is promising. The observed errors are most likely due to a combination of lack of knowledge on the glucose regulatory network and simplifications required by the Boolean formalism. In this regard it should be noted that the deterministic and binary approximation to reality inherent in the Boolean formalism demands careful interpretation of model outputs and limits the overall success rate, which may be achieved. Even though there is much information on glucose repression in yeast, it is clear from our analysis that there are still connections and parts that are missing. Since the model is set up rigorously based on data from the literature, these inconsistencies seem to be caused by a combination of contradictions in reported experimental results and perhaps due to incorrect insights about the network topology. Future efforts in modeling of glucose repression may need to take into consideration the uncertainties concerning the connectivity of the regulatory network, as well as network dynamics. Methods to discern these regulatory uncertainties (for example, through the identification of the most probable regulatory pathway from a set of different mechanistic pathway models), moreover have the potential to be used for reverse engineering of signaling and regulatory networks. Nevertheless, the model presented here represents a condensed way of organizing regulatory information on glucose repression, and strongly facilitates integration and evaluation of new hypotheses. It can also serve as the basis for further efforts in modeling glucose repression signaling and regulatory pathways using probabilistic and/or dynamic approaches. The model presented here is thus an important step towards a holistic understanding of glucose repression in *Saccharomyces cerevisiae*, and the model may further be used for design of new experiments that can lead to a better understanding of this complex regulatory system.

## Methods

### Network reconstruction and logical model representation

Glucose repression signaling and regulatory network was reconstructed from low-throughput data, namely from biochemical studies and physiological observations reported in peer-reviewed, original research publications. All information found relevant regarding glucose repression regulatory cascades was collected in a database specifying the species involved (genes, proteins and metabolites) and the type of regulation exerted among them. This information was then converted into a logical hypergraph, representing all interactions between species in a logical manner, according to the framework proposed by *Klamt et al*. [17]. In our context, a hypergraph is a generalized unipartite directed graph representation of an interaction network where each edge (also called hyperarc or hyperedge) connects a set of start-nodes (tails) to a set of end-nodes (heads). Here, we consider graphs with one or more start-nodes, but with a single end-node. Nodes represent the different species (genes, proteins or metabolites) and hyperarcs represent the signal flow between species. A logical hypergraph is a hypergraph where hyperarcs are represented by Boolean (or logical) equations (Figure 2), meaning that the state of an end-node can be deterministically found from the state of start-nodes based on the defined Boolean function connecting these nodes. Nodes can assume one of two logical states, on (1) and off (0); a gene can be expressed (1) or not (0) (or, in a more specialized case, be upregulated (1) or expressed at a basal level (0)), a protein can be active (1) or not (0), a metabolite can be available (1) or not (0). Logical states represent a discrete approximation of a continuous reality, for example, a discrete approximation of the sigmoid curve dictated by the Hill equation used to describe both gene expression and enzyme activity. Furthermore, we notice that the hypergraph representation of a Boolean network requires all logical equations to be written in the so-called disjunctive normal form, which uses exclusively AND, OR and NOT operators [17] (Figure 2). All network interactions were therefore converted into Boolean functions written in disjunctive normal form, and logical rules were introduced based on literature information. The hereby reconstructed logical hypergraph can easily and unambiguously be converted to its underlying interaction graph by splitting up hyperarcs with more than one tail.

Our logical hypergraph model represents sensing events (metabolite – protein interactions), signaling cascades (protein – protein interactions) and regulatory circuits (protein – gene interactions) related with glucose repression in *S. cerevisiae*. For analyses purposes, we consider the sensing events (sugar availability) as the input layer of the system, while the expression levels of the gene nodes are the outputs used in the model evaluation. Thus, by properly defining an initial state of the input layer, we can determine the logical steady state of all nodes in the system given the defined set of logical rules underlying the hypergraph. Additionally, given an input node and an output node, we can also perform a number of structural analyses on the characteristics of the pathways connecting them. All our functions are time-independent, as we are only interested in logical steady state solutions. Logical steady states analyses simplify the hypergraph setup, since we do not need to take into consideration the different timescales of different processes. Moreover, it allows the model steady states solutions to be evaluated against data from steady state chemostat cultivation or from the exponential phase of a batch cultivation (where balanced growth resulting in appearance of pseudo-steady-state can be assumed).

### Structural and logical steady state analyses of the network

We used the MATLAB toolbox CellNetAnalyzer 7.0 [17,51] to perform structural and functional logical steady state analyses on the established network. Structural analyses (number of loops and dependency matrix) were performed on the underlying interaction graph derived from the hypergraph. We used CNA capabilities to analyze the overall number of positive and negative loops between all input nodes and output nodes. We also determined the dependency matrix, which summarizes the relationship between all *ordered pairs *of species in the network. The dependency matrix is based solely on the topology of the interaction matrix and does not incorporate information on Boolean relationships [17,51], i.e. while the dependency matrix may tell us that A is an activator of B, it does not tell us whether species C must be present for the activation to take place. Each matrix element, D_ij_, tells us whether the network contains (1) only activating paths, (2) only inhibiting paths, or (3) both activating and inhibiting paths, between species *i *and *j*. In addition, it tells us whether negative feedback loops exist that may attenuate the predicted (1) activating or (2) down-regulatory effects (if this is the case, species *i *is referred to as a *non-total *activator or inhibitor). The dependency matrix thus summarizes information on network topology in a very condensed way. It was particularly helpful in setting up the underlying interaction graph, and in identifying parts of the network that were inconsistent with information in the literature.

Logical steady state calculations were performed based on the logical hypergraph representation. Briefly, the logical steady state is the Boolean state that the system eventually reaches given a fixed input (see [17] for detail). We used CNA to determine the logical steady state of all nodes in the system under all logical combinations of sugars availability (glucose, galactose, and/or maltose), and for all single gene deletions and some double gene deletions. In general, all nodes (except the input layer) are by default unconstrained. A few species were given a default value of 1 (genes where no other regulation is considered and genes expressed at basal levels). Gene deletions (i.e., knockouts) were simulated by setting the state of the deleted gene to a fix value of 0. Finally, specific edges were ignored in logical steady state analysis if the corresponding regulatory interactions were comparably weak.

### Model evaluation for knockouts

For some of the knockouts we were able to evaluate model predictions with available gene expression data from transcriptome studies. We used the results from the logical steady state analyses for the corresponding conditions in order to calculate the changes in gene expression between the simulated wild-type and the simulated knockout mutant. For wild-type simulations we obtained, for each species *i*, a Boolean state X_i,WT _∈ {0,1}. Similarly, each knockout simulations produces a logical state X_i,KO _∈ {0,1}. The variation between these two conditions is given by the difference Y_i_^mod ^= (X_i,KO _- X_i,WT_), with Y_i_^mod ^∈ {-1,0,1}. If species *i *is a gene (transcript), then Y_i_^mod ^can be compared with experimental differential gene expression data, and such comparison allow us to evaluate the predictive capability of the model. Thus, we used the available transcriptome data in order to convert experimental gene expression changes for each gene *i *into a discrete number Y_i_^exp ^∈ {-1,0,1}, based on their significance of change (*p*-value from a Student's *t*-test) and fold change (defined as FC = "average expression of gene *i *in knockout"/"average expression of gene *i *in wild-type" if "average expression of gene *i *in knockout" = "average expression of gene *i *in wild-type", otherwise FC = -1 × "average expression of gene *i *in wild-type"/"average expression of gene *i *in knockout"). We established a fold-change threshold ([FC_min_| = 1.5) and a Student's *t*-test *p*-value cut-off (α = 0.05) for all pair-wise gene expression comparisons between a deletion mutant and its isogenic reference strain (i.e., wildtype). All genes with *p*-value < α and FC ≥ FC_min _(or FC ≤ -FC_min_) were assigned with a value of Y_i_^exp ^= 1 (or Y_i_^exp ^= -1), and 0 otherwise. Overall, the model prediction capabilites were evaluated based on the difference |Y_i_^mod ^- Y_i_^exp^|, a value of 0 meaning a correct prediction, a value of 1 implying a small error, and a value of 2 indicating a large error (model prediction and experimental results in opposite directions).

### Evaluation of predictive power

The capabilities of the model to make predictions were evaluated in terms of the achieved percentage of correct predictions and by testing the results against the values expected from a model making random predictions. For each knockout evaluation, the ratio of correct predictions was calculated as the ratio of the number of genes where |Y_i_^mod ^- Y_i_^exp^| = 0, divided by the total number of genes evaluated against experimental gene expression data. In order to remove uncertainty, we also determined the percentage of correct predictions excluding dubious interactions. The model predictions were statistical evaluated in order to assess the probability of having correct predictions by chance, a high number indicating a very bad predictive model. In order to do so, our model predictions were tested against a random model using the normal approximation to the binomial distribution. Specifically, using a one-sided alternative, we tested the null hypothesis that the proportion of correct predictions by the model, p = X/n (X being the number of successes and n being the total sample size, i.e. the number of genes tested), is equal to that expected from the random model, p_0_. The statistic used is:

$$Z=\frac{X-np_{0}}{\sqrt{np_{0}(1-p_{0})}}$$

which is a random variable approximated by the standard normal distribution [52].

## Abbreviations

ODE: ordinary differential equations; CNA: CellNetAnalyzer (MATLAB toolbox).

## Authors' contributions

TSC reconstructed the network, performed all the analyses, and contributed to the writing of the manuscript. APO conceived, designed and supervised the study, was involved in discussing results, and contributed to the writing of the manuscript. JN designed and coordinated the study. All authors read and approved the final manuscript.

## Supplementary Material

Additional file 1 **Commented list of regulatory species and interactions included in the Boolean model**. Table containing all regulatory species considered, a description of their function, their mode of regulation and respective references, and, in some cases, additional notes. This table constitutes the basis for the Boolean associations used in the Boolean model. Click here for file

Additional file 2 **Logical equations included in the hypergraph**. Table containing all logical equations included in the computational evaluation of the hypergraph. In some cases, the logical equations are accompanied by a note. Click here for file

Additional file 3 **Evaluation of the logical state of the system for all gene deletions and different carbon sources availability.** The Excel file sheet 'KO-WT' contains the evaluation of the model prediction Y_i_^mod ^(Y_i_^mod ^= X_i,KO_^mod ^- X_i,WT_^mod^) for all single gene deletions and few double deletions under all combinations of available carbon sources used in this study. The sheet 'WT_copy' contains the state of the system for the wild-type (X_i,WT_^mod^) under all combinations of carbon sources. The sheet '2KO_Evalution' contains the state of the system for all gene deletions (X_i,KO_^mod^) under all combinations of carbon sources. Click here for file
