# Supplementary material for: Reconstruction and logical modeling of glucose repression signaling pathways in Saccharomyces cerevisiae
Source: BMC Syst Biol. 2009 Jan 14;3:7. doi: 10.1186/1752-0509-3-7 (PMC2661888; doi:10.1186/1752-0509-3-7)
Supplement: Additional file 1 — Commented list of regulatory species and interactions included in the Boolean model. Table containing all regulatory species considered, a description of their function, their mode of regulation and respective references, and, in some cases, additional notes. This table constitutes the basis for the Boolean associations used in the Boolean model. [file 1752-0509-3-7-S1.pdf]

Supplementary Information

Reconstruction and logical modeling of glucose repression signaling pathways in *Saccharomyces cerevisiae*

Tobias S Christensen, Ana Paula Oliveira, Jens Nielsen

Additional File 1. Commented List of Species and Interactions

Species (gene name)	Function of gene product	Regulators	Reference concerning regulation	Notes on Species
SNF3	Plasma membrane glucose sensor, hexotransporter homologue	Activated by glucose on the protein level	(Johnston and Kim, 2005; Kaniak et al., 2004; Kim et al., 2006; Kim and Johnston, 2006; Polish et al., 2005)	Probably a high affinity glucose sensor expressed in low glucose concentrations (Kaniak et al., 2004). The present model for the role of Snf3 and Rgt2 is as follows (Kim et al., 2006): Mth1 and Std1 interact with the C-terminal domains of Snf3 and Rgt2 placing within reach of the Yck1 kinase. In the presence of glucose Mth1 and Std1 are phosphorylated and marked for degradation by the SCF^{Grr1} ubiquitin ligase.
		Repressed by Mig1	(Kaniak et al., 2004)	
		Repressed by Mig2	(Kaniak et al., 2004)	
		Repressed by Mig3 (weakly)	(Kaniak et al., 2004)	
RGT2	Plasma membrane glucose sensor, hexotransporter homologue	Activated by glucose on the protein level	(Johnston and Kim, 2005; Kaniak et al., 2004; Kim et al., 2006; Kim and Johnston, 2006; Polish et al., 2005)	See note on *SNF3*
***YCK*1/2**	Casein kinase 1	None in model	(Moriya and Johnston, 2004; Schmidt et al., 1999)	Palmitoylated, plasma membrane-bound protein kinase involved in diverse gene functions. Encoded in two isoformes by *YCK*1 and *YCK*2. At least one of the two isoforms is required for viability. In the present model, *YCK*1/2 is active per default.
GRR1	F-box protein, component of the SCF ubiquitin ligase	None in model	(Moriya and Johnston, 2004)	Grr1 is a specificity factor (i.e. the F-box protein) for the class of ubiquitin ligase complexes SKp1/cullin/F-box protein (SCF). In the present model, the SCF ubiquitin ligase having Grr1 as specificity factor is refered to as SCF-Grr1. This complex is, additionally to glucose repression, involved in among other things cell cycle control. In the present model, *GRR1*, as well as the protein complex it forms with the SCF ubiquitin ligase, is active per default. (The other, general components of the SCF have not been included at the level of

Species (gene name)	Function of gene product	Regulators	Reference concerning regulation	Notes on Species
				the genes)
MTH1	Regulator of Rgt1 activity	Repressed by Mig1	(Kaniak et al., 2004)	Negative regulator of hexotransporters, required for transcriptional repression by Rgt1. Interacts with the C-terminal cytosolic tails of Snf3 and Rgt2.
		Repressed by Mig2	(Kaniak et al., 2004)	
		Repressed by Mig3 (weakly)	(Kaniak et al., 2004)	
		Weakly repressed by Rgt1	(Kaniak et al., 2004)	
		Weakly activated by Gal4	(Kaniak et al., 2004)	
		Mth1 protein degradation at the proteasome	(Flick et al., 2003; Kim et al., 2006; Moriya and Johnston, 2004; Polish et al., 2005)	The degradation (like for Std1) is mediated in the presence of glucose through subsequent interactions with Rgt2 or Snf3, Yck1 and SCF-Grr1.
STD1	Regulator of Rgt1 activity	Repressed by Rgt1	(Kaniak et al., 2004)	Negative regulator of hexotransporters, required for transcriptional repression by Rgt1. Interacts with the C-terminal cytosolic tails of Snf3 and Rgt2. Highly homologous with Mth1 with apparently partly overlapping functions.
		Std1 protein degradation at the proteasome	(Flick et al., 2003; Kim et al., 2006; Moriya and Johnston, 2004; Polish et al., 2005)	Degradation of Std1 (like Mth1) is mediated in the presence of glucose through subsequent interactions with Rgt2 or Snf3, Yck1 and SCF-Grr1.
RGT1	Transcription factor	Mth1 or Std1 requred for transcriptional repression by Rgt1	(Kim et al., 2006; Kim and Johnston, 2006; Ozcan et al., 1996)	Rgt1 seems to be the primary regulator of a number of hexotransporter as well as being a regulator of Mig2 (Kaniak et al., 2004. The primary role of Rgt1 seems to be as a repressor, however, it also seems to be involved in the activation of its target genes in the presence of glucose (Kim et al., 2006; Kim and Johnston, 2006; Ozcan et al., 1996) - This however, has not been incorporated into the model, as derepression (as opposed to activation) is sufficient for significant target gene expression (and this is, as will hopefully be apparent by now, a Boolean model). In this model, besides from being corepressors of Rgt1, Std1 and Mth1 have also been given the role as activators of the Rgt1 protein. The reason for this is that in some cases, like that of Mig2, it is not clear whether Rgt1 repression requires the presence of both Mth1 and Std1, the presence of either one of the two, or e.g. only Mth1 - however it is generally acknowledged that Rgt1 does not act as a repressor without one of the two.
				In addition, it should be noted that Rgt1, like the Mig proteins, functions as a repressor via the recruitment of the general transcription factors Tup1

Species (gene name)	Function of gene product	Regulators	Reference concerning regulation	Notes on Species
				and Ssn6 (also known as Cyc8). - These two proteins have not been included into the present model due to their ubiquitous nature in this type of model. Finally it has been shown that Rgt1 is also mediated through phosphorylation (Kim and Johnston, 2006; Santangelo, 2006), possibly through the action of protein kinase A (encoded by the TPK1-3 genes). - This, however, has not been included in the present model, for the same reason that Snf1 regulation was not more thoroughly described, i.e. lack of information.
GLC7* and *REG1	Glc7 is protein phosphatase 1, Reg1 is the regulatory subunit that targets Glc7 to dephosphorylation of Snf1	Indirectly activated towards Snf1 by glucose.	(Carlson, 1999; Rolland et al., 2002; Santangelo, 2006)	The regulation of the Snf1 protein kinase in response to glucose is complex and not fully understood and is therefore not explicitly included the present model. The most favoured model has been reviewed recently by (Santangelo, 2006). It apparently involves activation of adenylate cyclase, Cyr1, through either of two pathways, one involving the G-protein coupled receptor Gpr1 and its corresponding G-protein Gpa2, the other involving the Ras protein. Upon addition of glucose, these pathways leads to a transient increase in the cAMP and ensuing activation of protein kinase A (PKA, the catalytic subunits being encoded redundantly by TPK1-3). PKA then somehow induces hexokinase 2 (encoded by HXK2) to act on the Glc7/Reg1 complex. The final consequence of this is that the phosphatase activity of Glc7 is redirected towards Snf1. As a consequence, the Snf1 kinase is inactivated. Due to the ambiguities associated with this model, we here simply state that the Glc7/reg1 complex is activated (i.e. it becomes active towards Snf1) in the present of extracellular glucose.
SNF1* and *SNF4	Snf1 and Snf4 along with either Gal83p, Sip1p, or Sip2p forms a serine/threonine protein kinase complex.	Inhibited by dephosphorylation by the Glc7Reg1 complex	(Ludin et al., 1998; Santangelo, 2006)	The protein kinase complex (refered to in the model simply as Snf1) consists of the catalytic subunit encoded by SNF1, a regulatory subunit which is essential for Snf1 activity encoded by SNF4 as well as a third subunit encoded by either GAL83, SIP1 or SIP2. The present model includes only SNF1 and SNF4 since the function of Gal83, Sip1 and Sip2 is less clear, though they are probably important in regards to substrate specificity (Santangelo, 2006). The Snf1 protein kinase complex regulates the activity of several transcription factors including Mig1 and Sip4 (Rolland et al., 2002).The activity of Snf1 seems to be mediated primarily through the protein phosphatase complex Glc7-Reg1 (knockout of either GLC7 or REG1 yields a constitutively active Snf1 as well as by three protein kinases, Sak1, Tos3 and Elm1, which are not included in the model as their regulation and apparently overlapping function is poorly understood (knockout of all three results in a Δ*snf1* phenotype) (Santangelo, 2006).
MIG1	Transcription Factor	Repressed by Mig1	(Kaniak et al., 2004; Rolland et al., 2002)	The zinc finger protein Mig1 is arguably the most important transcription factor in glucose repression. It generally functions as a transcriptional repressor by recruitment of Tup1 and Ssn1 (like Rgt1 and the other Mig proteins), although there have been reports of it acting as a transcriptional activator (Lutfiyya et al., 1998).
		Repressed by Mig2	(Kaniak et al., 2004)	

Species (gene name)	Function of gene product	Regulators	Reference concerning regulation	Notes on Species
		Repressed by Mig3 (weakly)	(Kaniak et al., 2004)	
		Inhibited by phosphorylatio n by the Snf1/Snf4 protein kinase complex	(Rolland et al., 2002)	
MIG2	Transcription Factor	Repressed by Rgt1	(Kaniak et al., 2004)	MIG2 encodes a Mig1 homologue. Mig1 and Mig2 apparently have the same target genes, although the binding affinities may vary, but Mig2 as opposed to Mig1 is not regulated by the Snf1/Snf4 protein kinase (Lutfiyya and Johnston, 1996). The regulation of Mig2 is not fully understood, however, it is known to be regulated on the transcriptional level by Rgt1 (Kaniak et al., 2004)
MIG3	Transcription Factor	Repressed by Rgt1	(Kaniak et al., 2004)	MIG3 encodes a homologue of Mig1 and Mig2 and presumably have roughly the same gene targets as these two proteins. However, no physiologically significant role of Mig3 has yet been determined (Lutfiyya et al., 1998; Lutfiyya and Johnston, 1996; Santangelo, 2006).
		Inhibited by phosphoryla-tion by Snf1	(Dubacq et al., 2004)	
***MALR* (*MAL*23, *MAL*43, *MAL*63, *MAL*33, *MAL*13)**	Transcriptional activator of *MAL* genes	Repressed by Mig1	(Hu et al., 1995; Hu et al., 2000)	Maltose fermentation in Saccharomyces cerevisiae requires two enzymes, the maltose permease and maltase. The induction of these proteins are mediated through a regulatory protein (MALR). The three genes map to a three gene complexes known as the MAL loci, of which 5 are currently known, each on a different chromosome (Hu et al., 1995), e.g. MAL6 on chromosome VIII. Mig1 binding to the promoter of each MAL gene have been shown for the MAL6 locus (Hu et al., 1995), however, the investigation is complicated by the fact that e.g. the MAL23 (encoding the MAL activator protein, MalR) does not have a Mig1 binding site due to point deletion (Hu et al., 2000). The MAL regulatory system is thus quite complex and it should be emphathised that the model presented here is most likely a simplified version of the truth - e.g. all activators, maltases and permeases, respectively, are treated as one.
		Activated on the protein level by intracellular maltose	(Hu et al., 1995; Hu et al., 2000; Wang et al., 2002)	
***MALT* (*MAL*21, *MAL*41, *MAL*61, *MAL*31, *MAL*11)**	Maltose Permease	Repressed by Mig1	(Hu et al., 1995; Hu et al., 2000)	Transporter of maltose (essential for transport, the additional MAL genes are not induced if this is not active (Klein et al., 1998; Wang et al., 2002). Since deletion of all *MALT* genes prevents maltose induction, it seems reasonable to assume that MalT must be expressed at a basal level.
		Transcriptional ly activated by MalR	(Hu et al., 1995; Hu et al., 2000)	

Species (gene name)	Function of gene product	Regulators	Reference concerning regulation	Notes on Species
		Inhibited by glucose on the protein level (possibly indirectly)	(Hu et al., 1995; Hu et al., 2000)	
GAL1	Galactokinase, posttranslational regulator of Gal80	Repressed by Mig1	(Lohr et al., 1995; Nehlin et al., 1991)	Involved in high affinity galactose transport (Klein et al., 1998).
		Activated by Gal11	(Lohr et al., 1995)	
		Activated by Gal4	(Lohr et al., 1995)	
GAL2	Galactose permease	Activated by Gal4	(Lohr et al., 1995; Nehlin et al., 1991)	Required for utilization of galactose; also able to transport glucose (Klein et al., 1998).
		Catabolite inactivated by glucose	(Matern and Holzer, 1977)	
		Activated by Gal11	(Lohr et al., 1995)	
GAL3	Regulator that mediates galactose induced inhibition of Gal80	Activated by Gal4	(Lohr et al., 1995)	Regulator involved in activation of the GAL genes in response to galactose; forms a complex with Gal80 to relieve Gal80 inhibition of Gal4; binds galactose and ATP but does not have galactokinase activity. In the present model it is assumed that galactose must be taken up by the cell (i.e. the transporters must be active) for Gal3 to be induced.
		Repressed by Mig1	(Lundin et al., 1994; Verma et al., 2005)	
		Activated by galactose on the protein level (possibly indirectly)	(Lohr et al., 1995; Nehlin et al., 1991; Peng and Hopper, 2002)	
GAL4	Transcription factor, activator of GAL genes	Repressed by Mig1	(Lohr et al., 1995; Nehlin et al., 1991)	Transcriptional activator of the *GAL* gene family.
		Inhibited by Gal80 on the protein level	(Lohr et al., 1995; Peng and Hopper, 2002)	
GAL11	Transcription factor, part of the mediator complex	Supposed to be constantly active in the model		Component of the Mediator complex; interacts with RNA polymerase II and the general transcription factors to form the RNA polymerase II holoenzyme; affects transcription by acting as target of activators and repressors. Null mutant is viable, exhibits reduced expression of Gal4 regulated genes" (SGD). According to (Lohr et al., 1995) Gal11 probably forms a complex with Gal4
GAL80	Inhibitor of *GAL4* transcription factor	Activated by Gal4	(Lohr et al., 1995)	Forms complex with Gal4. In the absence of galactose, Gal80 masks the transcriptional activator capabilities of Gal4 even when this protein is bound to the promoter. The presence of galactose induces Gal3 to relieve the inhibitory effect of Gal80 on Gal4 by sequestering Gal80 to the cytosol (Peng and Hopper, 2002). Gal1 have also been reported to

Species (gene name)	Function of gene product	Regulators	Reference concerning regulation	Notes on Species
				inhibit Gal4, but since Gal1 is only expressed in the absence of Gal4, this is probably only important in maintaining the signal.
		Inhibited by Gal1 on the protein level	(Bhat and Hopper, 1992; Lohr et al., 1995)	
		Inhibited by Gal3 on the protein level	(Bhat and Hopper, 1992; Lohr et al., 1995)	
CAT8	Transcriptional activator	Repressed by Mig1	(Roth et al., 2004; Schuller, 2003; Vincent and Carlson, 1998)	Transcriptional activator involved in the regulation of glyconeogenic genes, the glyoxylate cycle and ethanol utilization.
		Presumably activated by Snf1 phosphorylatio n.	(Schuller, 2003; Vincent and Carlson, 1999)	
SIP4	Transcriptional activator	Activated by Cat8	(Roth et al., 2004; Schuller, 2003; Vincent and Carlson, 1998)	Homologue of Cat8, possibly with a more specific consensus sequence (Roth et al., 2004; Vincent and Carlson, 1998).
		Presumably activated by Snf1 phosphorylatio n.	(Schuller, 2003; Vincent and Carlson, 1999)	
**OUTPUT GENES**	**(i.e. the genes, of which the products are not part of the network)**			
SUC2	Invertase	Repressed by Mig1	(Klein et al., 1999; Lutfiyya and Johnston, 1996)	The invertase catalyzes the hydrolysis of sucrose to fructose and glucose. Acording to Klein et al (1999) the invertase actually increases significantly in Δ*mig1*, and a further increase is seen in Δ*mig1*Δ*mig2*. - Similar results have been found by Lutfiyya et al., although the effect of single knockouts was found to be less significant (Lutfiyya et al., 1998). According to (Zhou and Winston, 2001), Nrg1 is also required for glucose repression of SUC2 (this however is not incorporated into the model as the other articles show clear evidence that the effect is not significant - at least not under the conditions tested by Klein et al (1999) and Lutfiyya and Johnston (1996)).
		Repressed by Mig2	(Klein et al., 1999; Lutfiyya and Johnston, 1996)	
HXT1	Hexotransporter	Repressed by Rgt1 in the presence of	(Kaniak et al., 2004)	Low affinity glucose transporter, expressed when glucose is present in high concentrations (Rolland et al., 2002). Repression of this transporter apparently

Species (gene name)	Function of gene product	Regulators	Reference concerning regulation	Notes on Species
		either Std1 or Mth1		requires either Mth1 or Std1 aside from Rgt1 (Kim et al., 2006).
HXT2	Hexotransporter	Repressed by Rgt1	(Kaniak et al., 2004)	Regarded as an intermediate affinity glucose transporter according to (Rolland et al., 2002). It is not clear whether both Mth1 and Std1 are required for Rgt1 repression of this gene. Also repressed by Mig1 according to (Ozcan and Johnston, 1996).
		Repressed by Mig1	(Kaniak et al., 2004)	
HXT3	Hexotransporter	Repressed by Rgt1 in the presence of active Mth1	(Kaniak et al., 2004; Kim et al., 2006)	According to Rolland et al. (2002), a low affinity glucose transporter, expressed when glucose is present in high concentrations, though according to Kim et al. (2006) it is low glucose induced along with HXT4 (the latter statement seems to be the most well documented, see table 2 in Kim et al. (2006) – the gene is apparently induced at low glucose concentrations and its activity remains fairly constant in higher glucose concentrations – at least at the transcriptional level. Repression of this transporter apparently requires only Mth1 (not Std1, which does not assist in this repression) aside from Rgt1 (Kim et al.,2006). According to Özcan and Johnston (1996). HXT4 is probably also slightly repressed by Mig1, However, this interaction has not been included since the repression seems to be very weak.
HXT4	Hexotransporter	Repressed by Rgt1 in the presence of active Mth1	(Kaniak et al., 2004; Kim et al., 2006)	Regarded as a low-intermediate affinity glucose transporter according to Rolland et al (2002)
		Repressed by Mig1	(Ozcan and Johnston, 1999)	
HXT5	Hexotransporter	Repressed by Rgt1	(Kaniak et al., 2004)	Member of the Hexotransporter gene family. The repression by Rgt1 reported by (Kaniak et al., 2004) is probably by no means the only regulatory mechanism controlling this gene.
HXT8	Hexotransporter	Repressed by Rgt1	(Kaniak et al., 2004)	Member of the Hexotransporter gene family. The repression by Rgt1 reported by (Kaniak et al., 2004) is probably by no means the only regulatory mechanism controlling this gene.
YGL157 WYKR075C, YOR062C YNL234W	Putative open reading frames	Repressed by Rgt1	(Kaniak et al., 2004)	Open reading frames encoding unknown gene products, but which were found by (Kaniak et al., 2004) to be repressed by Rgt1.
MALS (***MAL22***, ***MAL*42**, ***MAL*62**, ***MAL*32**, ***MAL*12**)	Maltase	Repressed by Mig1	(Hu et al., 1995; Hu et al., 2000)	
		Transcriptionally activated by MalR	(Hu et al., 1995; Hu et al., 2000)	

Species (gene name)	Function of gene product	Regulators	Reference concerning regulation	Notes on Species
GAL5	Phosphoglucomutase 2	Activated by Gal4	(Klein et al., 1998; Lohr et al., 1995)	Catalyzes the epimerization of glucose-1-p to glucose-6-p. The expression pattern of this gene differs somewhat from the glucose repressed - galactose induced pattern seen for the other GAL genes, making it difficult to incorporate in the model (Lohr et al., 1995).
GAL7	Galactose-1-phosphate uridyl transferase	Activated by Gal4	(Lohr et al., 1995; Nehlin et al., 1991)	
GAL10	UDP-glucose-4-epimerase	Activated by Gal4	(Lohr et al., 1995; Nehlin et al., 1991)	Catalyzes the interconversion of UDP-galactose and UDP-D-glucose in the Leloir pathway
MEL1	Melibiase, secreted galactosidase	Activated by Gal4	(Lohr et al., 1995; Ostergaard et al., 2000)	(Lundin et al., 1994) showed that Mig1 binds to p-*MEL1* and although no direct experimental methods has verified Mig1 repression (as far as the author of this text is aware), a mathematical model by (Verma et al., 2005) incorporating Mig1 repression showed good correspondence with experimental data. *MEL1* is expressed in glycerol (Lohr et al., 1995), explained in the current model model via an OR relationship between Gal4 activation and Mig1 repression.
		Repressed by Mig1 (putatively)	(Lundin et al., 1994; Verma et al., 2005)	
ICL1	Isocitrate lyase	Activated by Cat8	(Roth et al., 2004; Schuller, 2003)	Catalyzes the formation of succinate and glyoxylate from isocitrate in the glyoxylate shunt.
		Activated by Sip4	(Roth et al., 2004; Schuller, 2003)	
FBP1	Fructose-1,6-bisphosphatase	Activated by Cat8	(Roth et al., 2004; Schuller, 2003)	Catalyzes the conversion of Fructose-1,6-bisphosphate to Fructose-6-phosphate in gluconeogenesis.
		Activated by Sip4	(Roth et al., 2004; Schuller, 2003)	
PCK1	Phosphoenolpyruvate carboxykinase	Activated by Cat8	(Roth et al., 2004)	Catalyzes the decarboxylation of oxaloacetate to yield PEP via ATP consumption in the gluconeogenesis.
MLS1	Malate synthase	Activated by Cat8	(Roth et al., 2004; Schuller, 2003)	Synthesizes Malate from glyoxylate and AcCoA in the glyoxylate shunt.
		Activated by Sip4	(Roth et al., 2004; Schuller, 2003)	
MDH2	Cytoplasmic malate dehydrogenase	Activated by Cat8	(Roth et al., 2004; Schuller,	Isoenzymes that catalyze interconversion of malate and oxaloacetate; involved in gluconeogenesis during growth on ethanol or acetate.

Species (gene name)	Function of gene product	Regulators	Reference concerning regulation	Notes on Species
			2003)	
		Activated by Sip4	(Roth et al., 2004; Schuller, 2003)	
ACS1	Acetyl-coA synthetase isoenzyme	Activated by Cat8	(Roth et al., 2004)	
SFC1	Succinate-fumarate mitochondrial transporter	Activated by Cat8	(Roth et al., 2004)	
CAT2	Carnitine acetyltransferase isoenzyme	Activated by Cat8	(Roth et al., 2004)	Involved in the import of activated acetate into the mitochondria (Schuller, 2003).
IDP2	Cytoplasmic, NADP dependent isocitrate dehydrogenase	Activated by Cat8	(Roth et al., 2004)	Catalyzes the conversion of Isocitrate into alpha-ketoglutarate
JEN1	Lactate permease	Activated by Cat8	(Roth et al., 2004)	

References for Supplementary Information

Bhat PJ and Hopper JE. Overproduction of the GAL1 or GAL3 protein causes galactose-independent activation of the GAL4 protein: evidence for a new model of induction for the yeast GAL/MEL regulon.Mol.Cell Biol. 12: 2701-2707 (1992).

Carlson M. Glucose repression in yeast.Curr.Opin.Microbiol. 2: 202-207 (1999).

Dubacq C, Chevalier A, Mann C. The protein kinase Snf1 is required for tolerance to the ribonucleotide reductase inhibitor hydroxyurea.Mol.Cell Biol. 24: 2560-2572 (2004).

Flick KM, Spielewoy N, Kalashnikova TI, Guaderrama M, Zhu Q, Chang HC, Wittenberg C. Grr1-dependent inactivation of Mth1 mediates glucose-induced dissociation of Rgt1 from HXT gene promoters.Mol.Biol.Cell 14: 3230-3241 (2003).

Hu Z, Nehlin JO, Ronne H, Michels CA. MIG1-dependent and MIG1-independent glucose regulation of MAL gene expression in Saccharomyces cerevisiae.Curr.Genet. 28: 258-266 (1995).

Hu Z, Yue Y, Jiang H, Zhang B, Sherwood PW, Michels CA. Analysis of the mechanism by which glucose inhibits maltose induction of MAL gene expression in Saccharomyces.Genetics 154: 121-132 (2000).

Johnston M and Kim JH. Glucose as a hormone: receptor-mediated glucose sensing in the yeast Saccharomyces cerevisiae.Biochem.Soc Trans. 33: 247-252 (2005).

Kaniak A, Xue Z, Macool D, Kim JH, Johnston M. Regulatory network connecting two glucose signal transduction pathways in Saccharomyces cerevisiae.Eukaryot.Cell 3: 221-231 (2004).

Kim JH, Brachet V, Moriya H, Johnston M. Integration of transcriptional and posttranslational regulation in a glucose signal transduction pathway in Saccharomyces cerevisiae.Eukaryot.Cell 5: 167-173 (2006).

Kim JH and Johnston M. Two glucose-sensing pathways converge on Rgt1 to regulate expression of glucose transporter genes in Saccharomyces cerevisiae.J Biol.Chem 281: 26144-26149 (2006).

Klein CJ, Olsson L, Nielsen J. Glucose control in Saccharomyces cerevisiae: the role of Mig1 in metabolic functions.Microbiology 144 (Pt 1): 13-24 (1998).

Klein CJ, Rasmussen JJ, Ronnow B, Olsson L, Nielsen J. Investigation of the impact of MIG1 and MIG2 on the physiology of Saccharomyces cerevisiae.J Biotechnol. 68: 197-212 (1999).

Lohr D, Venkov P, Zlatanova J. Transcriptional regulation in the yeast GAL gene family: a complex genetic network.FASEB J 9: 777-787 (1995).

Ludin K, Jiang R, Carlson M. Glucose-regulated interaction of a regulatory subunit of protein phosphatase 1 with the Snf1 protein kinase in Saccharomyces cerevisiae.Proc.Natl.Acad.Sci.U.S.A 95: 6245-6250 (1998).

Lundin M, Nehlin JO, Ronne H. Importance of a flanking AT-rich region in target site recognition by the GC box-binding zinc finger protein MIG1.Mol.Cell Biol. 14: 1979-1985 (1994).

Lutfiyya LL, Iyer VR, DeRisi J, DeVit MJ, Brown PO, Johnston M. Characterization of three related glucose repressors and genes they regulate in Saccharomyces cerevisiae.Genetics 150: 1377-1391 (1998).

Lutfiyya LL and Johnston M. Two zinc-finger-containing repressors are responsible for glucose repression of SUC2 expression.Mol.Cell Biol. 16: 4790-4797 (1996).

Matern H and Holzer H. Catabolite inactivation of the galactose uptake system in yeast.J Biol.Chem 252: 6399-6402 (1977).

Moriya H and Johnston M. Glucose sensing and signaling in Saccharomyces cerevisiae through the Rgt2 glucose sensor and casein kinase I.Proc.Natl.Acad.Sci.U.S.A 101: 1572-1577 (2004).

Nehlin JO, Carlberg M, Ronne H. Control of yeast GAL genes by MIG1 repressor: a transcriptional cascade in the glucose response.EMBO J. 10: 3373-3377 (1991).

Ostergaard S, Roca C, Ronnow B, Nielsen J, Olsson L. Physiological studies in aerobic batch cultivations of Saccharomyces cerevisiae strains harboring the MEL1 gene.Biotechnol.Bioeng. 68: 252-259 (2000).

Ozcan S and Johnston M. Two different repressors collaborate to restrict expression of the yeast glucose transporter genes HXT2 and HXT4 to low levels of glucose.Mol.Cell Biol. 16: 5536-5545 (1996).

Ozcan S and Johnston M. Function and regulation of yeast hexose transporters.Microbiol.Mol.Biol.Rev. 63: 554-569 (1999).

Ozcan S, Leong T, Johnston M. Rgt1p of Saccharomyces cerevisiae, a key regulator of glucose-induced genes, is both an activator and a repressor of transcription.Mol.Cell Biol. 16: 6419-6426 (1996).

Peng G and Hopper JE. Gene activation by interaction of an inhibitor with a cytoplasmic signaling protein.Proc.Natl.Acad.Sci.U.S.A 99: 8548-8553 (2002).

Polish JA, Kim JH, Johnston M. How the Rgt1 transcription factor of Saccharomyces cerevisiae is regulated by glucose.Genetics 169: 583-594 (2005).

Rolland F, Winderickx J, Thevelein JM. Glucose-sensing and -signalling mechanisms in yeast.FEMS Yeast Res. 2: 183-201 (2002).

Roth S, Kumme J, Schuller HJ. Transcriptional activators Cat8 and Sip4 discriminate between sequence variants of the carbon source-responsive promoter element in the yeast Saccharomyces cerevisiae.Curr.Genet. 45: 121-128 (2004).

Santangelo GM. Glucose signaling in Saccharomyces cerevisiae.Microbiol.Mol.Biol.Rev. 70: 253-282 (2006).

Schmidt MC, McCartney RR, Zhang X, Tillman TS, Solimeo H, Wolfl S, Almonte C, Watkins SC. Std1 and Mth1 proteins interact with the glucose sensors to control glucose-regulated gene expression in Saccharomyces cerevisiae.Mol.Cell Biol. 19: 4561-4571 (1999).

Schuller HJ. Transcriptional control of nonfermentative metabolism in the yeast Saccharomyces cerevisiae.Curr.Genet. 43: 139-160 (2003).

Verma M, Bhat PJ, Venkatesh KV. Steady-state analysis of glucose repression reveals hierarchical expression of proteins under Mig1p control in Saccharomyces cerevisiae.Biochem.J. 388: 843-849 (2005).

Vincent O and Carlson M. Sip4, a Snf1 kinase-dependent transcriptional activator, binds to the carbon source-responsive element of gluconeogenic genes.EMBO J 17: 7002-7008 (1998).

Vincent O and Carlson M. Gal83 mediates the interaction of the Snf1 kinase complex with the transcription activator Sip4.EMBO J 18: 6672-6681 (1999).

Wang X, Bali M, Medintz I, Michels CA. Intracellular maltose is sufficient to induce MAL gene expression in Saccharomyces cerevisiae.Eukaryot.Cell 1: 696-703 (2002).

Zhou H and Winston F. NRG1 is required for glucose repression of the SUC2 and GAL genes of Saccharomyces cerevisiae.BMC.Genet. 2: 5- (2001).
