# Supplementary material for: Reconstruction and logical modeling of glucose repression signaling pathways in Saccharomyces cerevisiae
Source: BMC Syst Biol. 2009 Jan 14;3:7. doi: 10.1186/1752-0509-3-7 (PMC2661888; doi:10.1186/1752-0509-3-7)
Supplement: Additional file 2 — Logical equations included in the hypergraph. Table containing all logical equations included in the computational evaluation of the hypergraph. In some cases, the logical equations are accompanied by a note. [file 1752-0509-3-7-S2.pdf]

Supplementary Information

Reconstruction and logical modeling of glucose repression signaling pathways in *Saccharomyces cerevisiae*

Tobias S Christensen, Ana Paula Oliveira, Jens Nielsen

Additional File 2. Logical Equations

The applied logical equations are summarized and (when deemed relevant) commented below. Each equation corresponds to a hyperedge. More detailed references are given in Additional file 1. Cells marked in yellow indicate that the equation is not considered in the computation of logical steady state. In very few cases, for reactions ignored in logical steady state calculations, a coefficient of 2 is used. These edges lead to species with default values of 1. The coefficient 2 is used in the hope that it will at some time be possible to implement in CNA that a gene is always expressed at a basal level (1), but may be induced to a higher level (2). A note about the nomenclature: genes are referred in uppercase (e.g., MIG1), while proteins include ths suffix 'p' (e.g., Mig1p). In the main text, proteins are referred similarly but without the suffix (e.g., Mig1), and genes are in italic uppercase (e.g., MIG1).

Logical Equation	Notes on logical equations
1 glucose_ext + 1 SNF3 = 1 Snf3p	In this version Snf3 is assumed to be inactive under high concentrations of glucose due to transcriptional repression. This may be a too drastic interpretation. However, since basal level gene expression is difficult to represent in CNA, the only alternative would be to give *SNF3* a default value of 1, which would mean that the model could not me used to make predictions of changes in *SNF3* expression levels
1 !Mig1p + 1 !Mig2p = 1 SNF3	It is assumed that Mig1 or Mig2 alone is sufficient to repress *SNF3*
1 !Mig3p = 1 SNF3	Ignored for logical steady state calculations as Mig3 is not physiogically relevant
1 glucose_ext + 1 RGT2 = 1 Rgt2p	
1 YCK1/2 = 1 Yck1p	
1 GRR1 = 1 SCF_Grr1	
1 !Mig1p = 1 MTH1	It is not entirely clear to the author whether Mig2 is a significant factor in the repression of *MTH1*. Here it is supposed that either Mig1 or Mig2 should be inactive for the gene to be derepressed
1 !Mig2p = 1 MTH1	
1 !Mig3p = 1 MTH1	Ignored for logical steady state calculations as Mig3 is not physiogically relevant
1 !Rgt1p = 1 MTH1	Ignored in logical computations, cf. Kaniak et al. (2004)
1 Gal4p = 1 MTH1	Ignored in logical computations, cf. Kaniak et al. (2004)
1 MTH1 + 1 !Yck1p = Mth1p	
1 MTH1 + 1 !SCF_Grr1 = Mth1p	
1 MTH1 + 1 !Snf3p + 1 !Rgt2p = Mth1p	
1 !Rgt1p = 1 STD1	Ignored in logical computations since *STD1* is active per default

Logical Equation	Notes on logical equations
1 STD1 + 1 !Yck1p = Std1p	
1 STD1 + 1 !SCF_Grr1 = Std1p	
1 STD1 + 1 !Snf3p + 1 !Rgt2p = Std1p	
1 Mth1p + 1 RGT1 = 1 Rgt1p	Rgt1 only function as repressor when Mth1 OR Std1 is present. *RGT1* is active per default
1 Std1p + 1 RGT1 = 1 Rgt1p	Rgt1 only function as repressor when Mth1 OR Std1 is present
1 glucose_ext + 1 REG1 + 1 GLC7 = 1 Glc7Reg1	
1 !Glc7Reg1 + 1 SNF1 + 1 SNF4 = 1 Snf1p	
1 !Mig2p =2 MIG1	Ignored in logical computations: Mig1 and Mig2 are active at the same time. We thus expect Mig1 to be most active while it is repressed at the transcriptional level
1 !Mig3p =2 MIG1	Ignored for logical steady state calculations as Mig3 is not physiogically relevant
1 !Snf1p + 1 MIG1 = 1 Mig1p	
1 !Rgt1p = 1 MIG2	
1 MIG2 = 1 Mig2p	Assuming that Mig2 is not post-translationally regulated.
1 !Rgt1p = 1 MIG3	
1 MIG3 + 1 !Snf1p = Mig3p	Assuming that the repression by Rgt1 is significant
1 !Mig1p = 1 MALR	
1 maltose_int + 1 MALR = 1 MalRp	
1 !Mig1p + 1 MalRp = 2 MALT	Ignored in logical computations since *MALT* is active per default: This is chosen as a consequence of the setup of CNA rather than due to physiological data: There must be a basal level of *MALT* expression for the logical steady state analysis to reach a result, for which reason *MALT* is given the default value of 1. However, given this default value, *MALT* can never reach the 2 level, for which reason this edge will be ignored in any case.
1 MALT = 1 MalTp	
1 !glucose_ext = 1 MalTp	Assuming that MalT is inhibited by glucose, either directly or indirectly, on the protein level. It is not obvious that this interaction should be excluded in logical computations, and further elucidation is required to decide in the future whether to include this equation. The choice did not, however effect the result of the present evaluations in the present report
1 !Mig1p + 1 Gal4p = 1 GAL1	This choice is not obvious. The reader is referred to Lohr et al. (1995): Significant repression by each individual mechanism, but only total repression when all repression pathways are active
1 Gal11p = 1 GAL1	Excluded in logical computations: Gal11 probably forms a complex with Gal4. Δ*gal11* diminishes Gal1 and Gal2 expression 10-11 times. However, a significant galactose induction would still be seen
1 GAL1 = 1 Gal1p	
1 Gal4p = 2 GAL2	In the present model, a basal level of Gal2 must be assumed, since intracellular galactose is necessary for the induction of Gal3
1 !glucose_ext = 1 Gal2p	The extent of glucose inhibition of galactose transport is not obvious based on literary data
1 Gal11p = 2 GAL2	Excluded in logical computations: Gal11 probably forms a complex with Gal4. Δ*gal11* diminishes Gal1 and Gal2 expression 10-11 times. However, a significant galactose induction would still be seen
1 GAL2 = 1 Gal2p	
1 Gal4p = 1 GAL3	Ignored in logical computations: If Mig1 is present Gal3 is not expressed. When grown on glycerol, Gal3 is present even though Gal4 is not active
1 !Mig1p = 1 GAL3	
1 GAL3 + 1 galactose_int = 1 Gal3p	

Logical Equation	Notes on logical equations
1 !Mig1p = 1 GAL4	
1 GAL4 + 1 !Gal80p = Gal4p	
1 GAL11 = 1 Gal11p	
1 Gal4p = 2 GAL80	This interaction cannot not be critical for the expression of Gal80, since Gal4 is only active when Gal80 is not present. Additionally, we must include a basal level of expression from *GAL80* by setting a default value of 1, so this edge would in any case be ignored in the logical steady state calculation
2 !Gal1p + 1 !Gal3p + 1 GAL80 = Gal80p	Here it is stated that Gal1 must be present at level 2 (which it never reaches in the wild type) to inhibit Gal80. This is done to stress that Gal1 over-expression can complement a *GAL3* deletion, but that Gal1 is probably only plays a minor role in Gal80 inhibition in the WT. Additionally, applying simply a 1 !Gal1p + 1 !Gal3p relationship would result in ambiguities in terms of multiple possible logical steady state solutions for several of the products of the *GAL* gene family
1 !Mig1p = 1 CAT8	
1 CAT8 + 1 Snf1p = 1 Cat8p	
1 Cat8p = 1 SIP4	
1 SIP4 + 1 Snf1p = 1 Sip4p	
1 !Mig1p + 1 !Mig2p = 1 SUC2	It is assumed that both Mig1 and Mig2 should be absent for SUC2 to be expressed at high levels, cf. Lutfiyya et al. (1998) who found that single deletions on had low impact on *SUC2* expression level, whereas Δ*mig1*Δ*mig2* had great effect**.** Contrary to this Klein et al. (1999) found a large increase in *SUC2* expression in a Δ*mig1* strain and further increase by additional disruption of *MIG2*
1 !Rgt1p = 1 HXT1	
1 !Mth1p + 1 !Std1p = HXT1	According to Kim et al. (2006), either Std1 or Mth1 is required for Rgt1 repression of *HXT1*.
1 !Mig1p + 1 !Rgt1p = 1 HXT2	Here it is assumed that Rgt1 can act as a repressor in the presence of either Mth1 OR Std1 (Mth1 and Std1 are not part of the expression since Rgt1 is only active if at least one of the two is present)
1 !Mth1p = 1 HXT3	
1 !Rgt1p = 1 HXT3	
1 !Mth1p + 1 !Mig1p = 1 HXT4	According to Kim et al. (2006), Mth1, but not Std1, is involved in Rgt1 repression of *HXT1*
1 !Rgt1p + 1 !Mig1p = 1 HXT4	
1 !Rgt1p = 1 HXT5	
1 !Rgt1p = 1 HXT8	
1 !Rgt1 = 1 4ORFs	
1 !Mig1p + 1 MalRp = 1 MALS	
1 Gal4p = 1 GAL5	Doubtable, see note on the species
1 Gal4 = 1 GAL7	
1 Gal4 = 1 GAL10	
1 Gal4p = 1 MEL1	
1!Mig1p = 1 MEL1	
1 Cat8p = 1 ICL1	Here it assumed that either Cat8 or Sip4 can activate gene expression. However, Sip4 expression is controlled by Cat8 in wild type cell
1 Sip4p = 1 ICL1	
1 Cat8p = 1 FBP1	Here it assumed that either Cat8 or Sip4 can activate gene expression. However, Sip4 expression is controlled by Cat8 in wild type cells
1 Sip4p = 1 FBP1	
1 Cat8p = 1 PCK1	
1 Cat8p = 1 MLS1	Here it assumed that either Cat8 or Sip4 can activate gene expression. However, Sip4 expression is controlled by Cat8 in wild type cells
1 Sip4p = 1 MLS1	

Logical Equation	Notes on logical equations
1 Cat8p = 1 MDH2	Here it assumed that either Cat8 or Sip4 can activate gene expression. However, Sip4 expression is controlled by Cat8 in wild type cells
1 Sip4p = 1 MDH2	
1 Cat8p = 1 ACS1	
1 Cat8p = 1 SFC1	
1 Cat8p = 1 CAT2	
1 Cat8p = 1 IDP2	
1 Cat8p = 1 JEN1	
1 galactose_ext + 1 Gal2p = 1 galactose_int	
1 maltose_ext + 1 MalTp = 1 maltose_int	

References for Supplementary Information

Bhat PJ and Hopper JE. Overproduction of the GAL1 or GAL3 protein causes galactose-independent activation of the GAL4 protein: evidence for a new model of induction for the yeast GAL/MEL regulon.Mol.Cell Biol. 12: 2701-2707 (1992).

Carlson M. Glucose repression in yeast.Curr.Opin.Microbiol. 2: 202-207 (1999).

Dubacq C, Chevalier A, Mann C. The protein kinase Snf1 is required for tolerance to the ribonucleotide reductase inhibitor hydroxyurea.Mol.Cell Biol. 24: 2560-2572 (2004).

Flick KM, Spielewoy N, Kalashnikova TI, Guaderrama M, Zhu Q, Chang HC, Wittenberg C. Grr1-dependent inactivation of Mth1 mediates glucose-induced dissociation of Rgt1 from HXT gene promoters.Mol.Biol.Cell 14: 3230-3241 (2003).

Hu Z, Nehlin JO, Ronne H, Michels CA. MIG1-dependent and MIG1-independent glucose regulation of MAL gene expression in Saccharomyces cerevisiae.Curr.Genet. 28: 258-266 (1995).

Hu Z, Yue Y, Jiang H, Zhang B, Sherwood PW, Michels CA. Analysis of the mechanism by which glucose inhibits maltose induction of MAL gene expression in Saccharomyces.Genetics 154: 121-132 (2000).

Johnston M and Kim JH. Glucose as a hormone: receptor-mediated glucose sensing in the yeast Saccharomyces cerevisiae.Biochem.Soc Trans. 33: 247-252 (2005).

Kaniak A, Xue Z, Macool D, Kim JH, Johnston M. Regulatory network connecting two glucose signal transduction pathways in Saccharomyces cerevisiae.Eukaryot.Cell 3: 221-231 (2004).

Kim JH, Brachet V, Moriya H, Johnston M. Integration of transcriptional and posttranslational regulation in a glucose signal transduction pathway in Saccharomyces cerevisiae.Eukaryot.Cell 5: 167-173 (2006).

Kim JH and Johnston M. Two glucose-sensing pathways converge on Rgt1 to regulate expression of glucose transporter genes in Saccharomyces cerevisiae.J Biol.Chem 281: 26144-26149 (2006).

Klein CJ, Olsson L, Nielsen J. Glucose control in Saccharomyces cerevisiae: the role of Mig1 in metabolic functions.Microbiology 144 (Pt 1): 13-24 (1998).

Klein CJ, Rasmussen JJ, Ronnow B, Olsson L, Nielsen J. Investigation of the impact of MIG1 and MIG2 on the physiology of Saccharomyces cerevisiae.J Biotechnol. 68: 197-212 (1999).

Lohr D, Venkov P, Zlatanova J. Transcriptional regulation in the yeast GAL gene family: a complex genetic network.FASEB J 9: 777-787 (1995).

Ludin K, Jiang R, Carlson M. Glucose-regulated interaction of a regulatory subunit of protein phosphatase 1 with the Snf1 protein kinase in Saccharomyces cerevisiae.Proc.Natl.Acad.Sci.U.S.A 95: 6245-6250 (1998).

Lundin M, Nehlin JO, Ronne H. Importance of a flanking AT-rich region in target site recognition by the GC box-binding zinc finger protein MIG1.Mol.Cell Biol. 14: 1979-1985 (1994).

Lutfiyya LL, Iyer VR, DeRisi J, DeVit MJ, Brown PO, Johnston M. Characterization of three related glucose repressors and genes they regulate in Saccharomyces cerevisiae.Genetics 150: 1377-1391 (1998).

Lutfiyya LL and Johnston M. Two zinc-finger-containing repressors are responsible for glucose repression of SUC2 expression.Mol.Cell Biol. 16: 4790-4797 (1996).

Matern H and Holzer H. Catabolite inactivation of the galactose uptake system in yeast.J Biol.Chem 252: 6399-6402 (1977).

Moriya H and Johnston M. Glucose sensing and signaling in Saccharomyces cerevisiae through the Rgt2 glucose sensor and casein kinase I.Proc.Natl.Acad.Sci.U.S.A 101: 1572-1577 (2004).

Nehlin JO, Carlberg M, Ronne H. Control of yeast GAL genes by MIG1 repressor: a transcriptional cascade in the glucose response.EMBO J. 10: 3373-3377 (1991).

Ostergaard S, Roca C, Ronnow B, Nielsen J, Olsson L. Physiological studies in aerobic batch cultivations of Saccharomyces cerevisiae strains harboring the MEL1 gene.Biotechnol.Bioeng. 68: 252-259 (2000).

Ozcan S and Johnston M. Two different repressors collaborate to restrict expression of the yeast glucose transporter genes HXT2 and HXT4 to low levels of glucose.Mol.Cell Biol. 16: 5536-5545 (1996).

Ozcan S and Johnston M. Function and regulation of yeast hexose transporters.Microbiol.Mol.Biol.Rev. 63: 554-569 (1999).

Ozcan S, Leong T, Johnston M. Rgt1p of Saccharomyces cerevisiae, a key regulator of glucose-induced genes, is both an activator and a repressor of transcription.Mol.Cell Biol. 16: 6419-6426 (1996).

Peng G and Hopper JE. Gene activation by interaction of an inhibitor with a cytoplasmic signaling protein.Proc.Natl.Acad.Sci.U.S.A 99: 8548-8553 (2002).

Polish JA, Kim JH, Johnston M. How the Rgt1 transcription factor of Saccharomyces cerevisiae is regulated by glucose.Genetics 169: 583-594 (2005).

Rolland F, Winderickx J, Thevelein JM. Glucose-sensing and -signalling mechanisms in yeast.FEMS Yeast Res. 2: 183-201 (2002).

Roth S, Kumme J, Schuller HJ. Transcriptional activators Cat8 and Sip4 discriminate between sequence variants of the carbon source-responsive promoter element in the yeast Saccharomyces cerevisiae.Curr.Genet. 45: 121-128 (2004).

Santangelo GM. Glucose signaling in Saccharomyces cerevisiae.Microbiol.Mol.Biol.Rev. 70: 253-282 (2006).

Schmidt MC, McCartney RR, Zhang X, Tillman TS, Solimeo H, Wolfl S, Almonte C, Watkins SC. Std1 and Mth1 proteins interact with the glucose sensors to control glucose-regulated gene expression in Saccharomyces cerevisiae.Mol.Cell Biol. 19: 4561-4571 (1999).

Schuller HJ. Transcriptional control of nonfermentative metabolism in the yeast Saccharomyces cerevisiae.Curr.Genet. 43: 139-160 (2003).

Verma M, Bhat PJ, Venkatesh KV. Steady-state analysis of glucose repression reveals hierarchical expression of proteins under Mig1p control in Saccharomyces cerevisiae.Biochem.J. 388: 843-849 (2005).

Vincent O and Carlson M. Sip4, a Snf1 kinase-dependent transcriptional activator, binds to the carbon source-responsive element of gluconeogenic genes.EMBO J 17: 7002-7008 (1998).

Vincent O and Carlson M. Gal83 mediates the interaction of the Snf1 kinase complex with the transcription activator Sip4.EMBO J 18: 6672-6681 (1999).

Wang X, Bali M, Medintz I, Michels CA. Intracellular maltose is sufficient to induce MAL gene expression in Saccharomyces cerevisiae.Eukaryot.Cell 1: 696-703 (2002).

Zhou H and Winston F. NRG1 is required for glucose repression of the SUC2 and GAL genes of Saccharomyces cerevisiae.BMC.Genet. 2: 5- (2001).
